# Supplementary material for: Adopting yield-improving practices to meet maize demand in Sub-Saharan Africa without cropland expansion
Source: Nat Commun. 2024 May 27;15:4492. doi: 10.1038/s41467-024-48859-0 (PMC11130130; doi:10.1038/s41467-024-48859-0)
Supplement: Supplementary file 1 — Supplementary Information [file 41467_2024_48859_MOESM1_ESM.pdf]

Supplementary Information for “Adopting yield-improving practices to meet maize demand in Sub-Saharan Africa without cropland expansion” by Aramburu-Merlos *et al*.

Table of Contents

Supplementary Notes 2
1. Agronomic Survey Questionnaire 2
2. Extrapolating the impact of yield-improving practices to Sub-Saharan Africa 2
    2.1. Direct extrapolation to the entire Sub-Saharan Africa: 2
    2.2. Machine learning extrapolation in regions with similar biophysical properties 2
Supplementary Tables 4
Supplementary Figures 6
Supplementary Code 43

List of Supplementary Tables

Table S1. List of survey-derived variables included in the analysis. 4
Table S2 List of environmental variables included in the analysis. 5

List of Supplementary Figures

Fig. S1. Seasonal weather patterns in the regions of study. 6
Fig. S2. Maize yield and main crop management practices in each country. 7
Fig. S3. Biophysical conditions in maize fields and Sub-Saharan Africa 8
Fig. S4. Dissimilarity between maize fields and Sub-Saharan Africa harvested area. 9
Fig. S5. Maize yield in Sub-Saharan Africa for two suites of management practices. 10
Fig. S6. Correlation coefficients of continuous variables. 11
Fig. S7. Correlation coefficients of categorical variables. 12
Fig. S8. Correlation coefficients between continuous and categorical variables. 13
Fig. S9. Association between key continuous and binary management variables. 14
Fig. S10. Proportion of soil variance captured by climate zones. 15
Fig. S11. Relative importance of factors explaining variation in maize yields. 16
Fig. S12. Conditional inference trees for each climate zone in the analysis 17

Supplementary Notes

1. Agronomic Survey Questionnaire

This survey was conducted by One Acre Fund (OAF) Monitoring and Evaluation (MEL) Team. At the end of each maize season, OAF MEL team members visited farmers and asked them for permission to measure maize yields and the final stands of plants in their fields. They also asked farmers whether they would answer some questions about the agronomic practices implemented in these maize fields.

Inform consent was obtained from all farmers providing information on their maize fields.

The questions were provided to OAF MEL team in English on a digital device. OAF MEL team asked them orally and recorded the answers from farmers in this device, translating any content as needed.

The questionnaire included the following questions and options.

1) How much land did you plant with maize this season?
2) What was the date that this maize field was planted?
3) What was the seed variety used? [choice][1]
    a. DK 777
    b. DK 8031
    c. DK 90-89
    d. H 614D
    e. H 6213
    f. H 6218
    g. PAN 691
    h. PN 15
    i. Pioneer P3812W
    j. SC DUMA 43
    k. SC PUNDA MILIA 53
    l. SC SIMBA 61
    m. SC Tembo 73
    n. WH 505
    o. WH 509
    p. SY594
    q. DK8033
    r. SC Sungura 301
    s. WH 513
    t. Don't Know
    u. Local Maize
    v. Other: [please specify]
4) How many kgs of Urea did you apply to the maize acres?
5) How many kgs of DAP did you apply to the maize acres?
6) How many kgs of CAN did you apply to the maize acres?

[1] The list of hybrids presented here was the one used in Kenya. Different hybrids were listed in each country to reflect the options available in each market.

7) How many kgs of NPK did you apply to the maize acres?

8) When you used fertilizer in this field, how did you determine what quantity to use? [choice]

   a. Used a spoon, or bottlecap, or other tool (not OAF Scoop)
   b. Used OAF Scoop
   c. Broadcast
   d. Did not use a tool
   e. I do not know/ I am not sure/ I don't remember

9) Did you apply compost and/ or manure this season to your plot? [choice]

   a. Yes - compost
   b. Yes - manure
   c. Both compost and manure
   d. No- I didn't apply compost or manure
   e. (If manure) from which animal?

10) What percentage of your field did you apply compost and/or manure to? [choice]

   a. 0% - 25%
   b. 25% - 50%
   c. 50% - 75%
   d. 75% - 100%

11) How much lime (kg) did you apply this season?

12) How many times did you weed this field?

13) Did you observe any of the following pest or disease problems in your maize field this season? (refer to images) Select all that apply

   a. MLND
   b. MSV
   c. Fall armyworm
   d. cutworms
   e. Stemborer
   f. Other pest/disease: please specify

14) Is striga present in this field? [choice]

   a. Yes
   b. No

15) Did you observe any flooding on this plot? [choice]

   a. Yes
   b. No

2. <u>Extrapolating the impact of yield-improving practices to Sub-Saharan Africa</u>

2.1. Direct extrapolation to the entire Sub-Saharan Africa:

To account for differences in biophysical background between the maize producing areas included in our field-level farmer database and that of the entire SSA, rather than simply assuming a same level of yield increase as the one shown in Fig. 5, we considered a scenario where farmers achieved the same level of yield gap (Yg) closure. The yield gap is the difference between average farmer yield and the yield potential (Yp), expressed relative to Yp. By expressing the yield increase relative to the Yp, we accounted for variation in Yp between regions.

The Yp of rainfed maize across the CZ included in our study was 10.6 t ha^{-1}, while the rainfed Yp across SSA maize producing areas is 10.2 t ha^{-1}. This subtle difference implies that a same level of Yg closure as the one achieved by the highest technology farmers in our data results in a slightly lower yield, as shown below:

$$\mathrm{Yg_{ht}} = 1 - \frac{\mathrm{Yht_{(SR)}}}{\mathrm{Yp_{(SR)}}} = 1 - \frac{4.3\ t\ ha^{-1}}{10.6\ t\ ha^{-1}} = 59\% \quad (1)$$

$$\mathrm{Yht_{(SSA)}} = \mathrm{Yp_{(SSA)}} \times (1 - \mathrm{Yg_{ht}}) = 10.2\ t\ ha^{-1} \times 41\% = 4.2\ t\ ha^{-1} \quad (2)$$

where Yp is the yield potential of rainfed maize, Yht is the yield of farmers adopting high technology, Yg is the yield gap farmers relative to the Yp, subscripts SR and SSA are for study region and Sub-Saharan Africa, respectively.

2.2. Machine learning extrapolation in regions with similar biophysical properties

We validated the estimation of average yield achieved with improved agronomic practices in SSA with a machine learning model. We trained Gradient Boosting Machine model with the field-level farmer data and several climatic and soil covariables (Table S1 and Table S2). We used this model to predict yields across SSA maize areas for two levels of management practices: baseline and intensified management. We constrained our predictions to rainfed maize areas with similar biophysical properties to those in our field-level observations. We included areas with a dissimilarity index < 40% (Fig. S4), representing 55% of SSA maize area. By doing so, we are accounting for differences in

biophysical background between our study area and other maize producing areas in SSA while ensuring meaningful predictions given the information provided to the machine learning model.

Site-specific results are shown in Fig. S5. The average yield of the baseline management (open pollination varieties, no fertilizer application, no weed or pest control, low plant densities, and medium to late sowing dates) was 1.9 t ha^{-1}, which is only *ca.* 100 kg ha^{-1} lower than the average maize yield in the entire SSA. In contrast, the machine learning model predicted an average maize yield of 4.2 t ha^{-1} for the intensified management practices (*i.e.,* highest technology level in Fig. 5), matching the value estimated by assuming a same level of yield gap closure in the entire SSA. Due to the high consistency between methods, we used the results from the "*direct extrapolation to the entire Sub-Saharan Africa*" (Supplementary Note 2.1) for the scenario assessment shown in Table 1, which considers the entire maize area in SSA, to emphasize the importance of agronomic improvement across the region.

Supplementary Tables

Table S1. List of survey-derived variables included in the analysis. The statistic column indicates the variable average ± its standard deviation (continuous variables), the percentage of positive (yes) answers (binary variables), or the percentage of observations in each category (categorical variables). Missing values were ignored to compute these quantities, but nil input rates were included. The NA column indicates the percentage of missing values for each variable.

Category	Name	Type	Unit	Description	Statistic	NA%*
Crop establishment	plant_date_dev	discrete	days	sowing date deviation from cluster average	0 ± 20	27
	pl_m2	continuous	# m^{-2}	plant density (plants per area)	3.7 ± 1.8	31
	row_spacing	continuous	cm	distance between rows	79 ± 12	44
Cultivar	hybrid	binary	-	Was a commercial hybrid seed used?	80%	8
	hyb_mat	ordinal	-	hybrid maturity (early, medium, late)	16-25-59	25
	hyb_yor	continuous	-	Year of release of the cultivar	2001 ± 7	50
	hyb_tol_mln	binary	-	Tolerance to maize lethal necrosis	2%	46
	hyb_tol_msv	binary	-	Tolerance to maize streak virus	22%	46
	hyb_tol_gls	binary	-	Tolerance to gray leaf spot	67%	46
	hyb_tol_nclb	binary	-	Tolerance to northern corn leaf blight	28%	46
	hyb_tol_rust	binary	-	Tolerance to rust	14%	46
	hyb_tol_ear_rot	binary	-	Tolerance to ear rot	51%	46
Nutrients	N_kg_ha	continuous	kg ha^{-1}	N fertilization rate	31 ± 29	0
	P_kg_ha	continuous	kg ha^{-1}	P fertilization rate	17 ± 14	0
	K_kg_ha	continuous	kg ha^{-1}	K fertilization rate	1.3 ± 5	25
	compost	binary	-	Was compost applied?	32%	40
	comp_t_ha	continuous	t ha^{-1}	compost rate	1 ± 4.2	54
	manure	binary	-	Did the compost contain manure?	78%	65
	fert_in_hole	binary	-	Was the fertilizer applied in a hole?	35%	0
Liming	lime_kg_ha	continuous	kg ha^{-1}	lime rate	7 ± 72	9
Adversities	weeding	discrete	#	number of times the plot was weeded	69%	29
	pesticide	binary	-	Was any pesticide applied?	35%	42
	disease	binary	-	Was yield affected by diseases?	3%	0
	pest	binary	-	Was yield affected by pests?	31%	0
	striga	binary	-	Was yield affected by the Striga weed?	34%	82
	water_excess	binary	-	Was yield affected by water excess (heavy rain or flooding)?	8%	0

* The percentage of missing values was calculated considering the observations for which an answer was expected (e.g., for hybrid maturity, considering only farmers who used hybrids)

Table S2. List of environmental variables included in the analysis.

Category	Name	Unit	Spatial resolution	Description	Source
Climatic*	GDD	°C days	30 arc-sec (1km)	Growing degree days	www.worldclim.org www.yieldgap.org
	AI	unitless	30 arc-sec (1km)	Aridity Index (annual precipitation over potential evapotranspiration)	
	TS	°C	30 arc-sec (1km)	Temperature seasonality	
Weather#	season_prec	mm	3 arc-min (5.6 km)	Total rainfall during the maize season (10% of planting to 50% of the harvest)	https://www.chc.ucsb .edu/data/chirps www.worldclim.org
	season_prec_1	mm	3 arc-min (5.6 km)	Rainfall during the first third of the season	
	season_prec_2	mm	3 arc-min (5.6 km)	Rainfall during the second third of the season	
	season_prec_3	mm	3 arc-min (5.6 km)	Rainfall during the last third of the season	
Elevation†	elev	m.a.s.l.	75 meters	Elevation (altitude) above sea level	registry.opend26ata. aws/terrain-tiles
Soil	soil_rzpawhc	mm	1 km	Root zone plant-available water holding capacity	www.isric.org
	soil_clay	%	30 meters	Clay content at 0-20cm soil depth	www.isda-africa.com
	soil_pH	-		pH (H_2O) at 0-20cm soil depth	
	soil_orgC	g/kg		Organic carbon at 0-20cm soil depth	
	soil_ECEC	$cmol_c$/kg		Effective cation exchange capacity at 0-20cm soil depth	
Topography	twi	unitless	75 meters	Topographic Wetness Index	calculated from elevation data

*Annual climatic variables were computed to delineate climate zones and crop season climatic conditions were considered for the dissimilarity index and machine learning model.

# Year-specific seasonal precipitation and year-specific precipitation in each third of the season was retrieved from CHIRPS and used in the conditional inference tree analysis, while long-term climatic average seasonal precipitation retrieved from WorldClim2 was used for results extrapolation with the Gradient Boosting Machine model.

†Elevation was used in the conditional inference tree analysis to account for fine environmental gradients within a CZ because of its high correlation with temperature and much higher resolution and accuracy than gridded temperature data. Due to differences in the relation between temperature and elevation across CZs, elevation was replaced with average temperature in the machine learning model.

Supplementary Figures

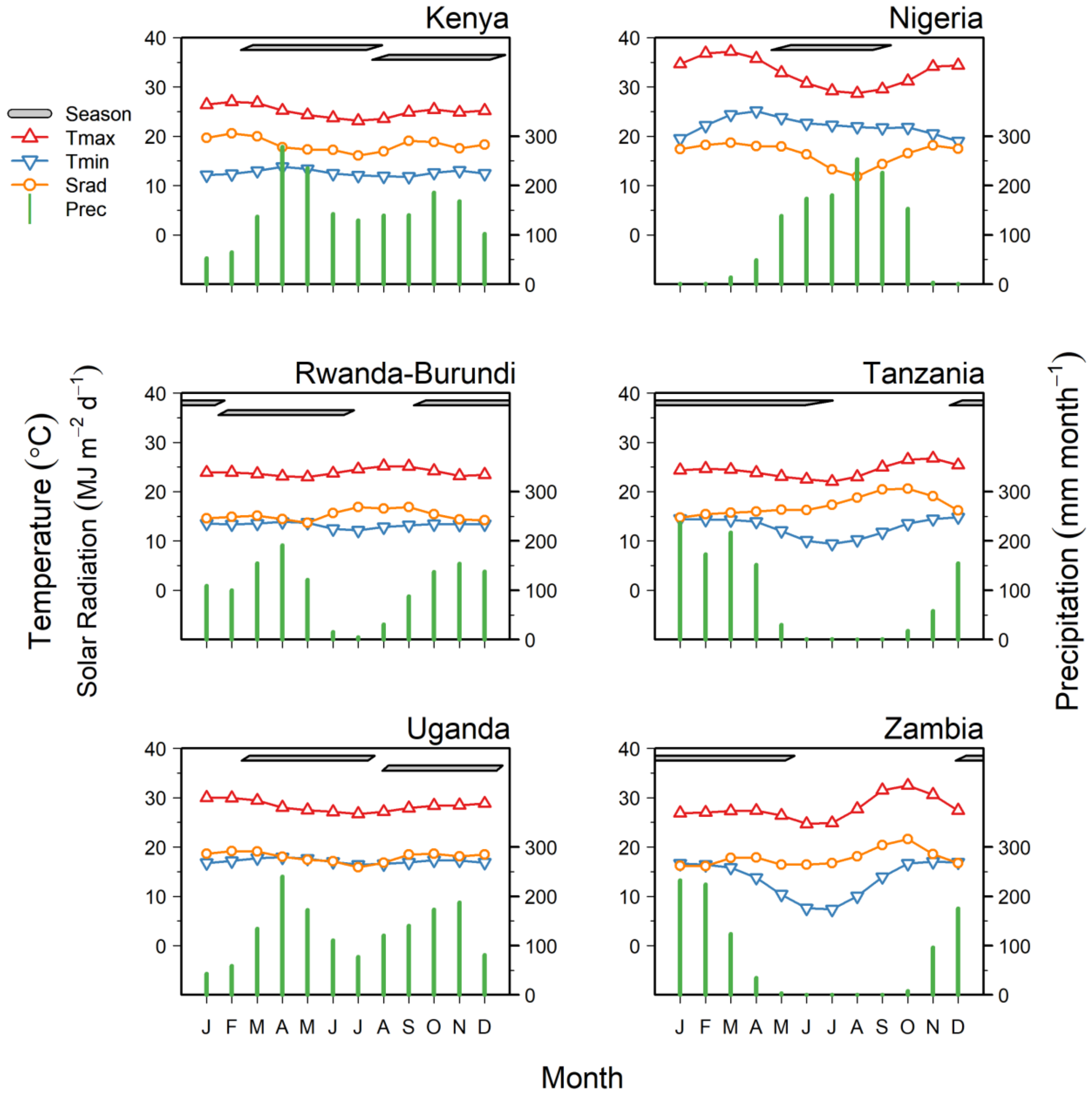

Fig. S1. Seasonal weather patterns in the regions of study. The horizontal parallelograms show maize growing seasons. There are two seasons per year in Kenya, Rwanda, Burundi, and Uganda (left panels), and a single season in Nigeria, Tanzania, and Zambia (right panels). Second season sowing and harvest dates in Kenya and Uganda were retrieved from the Global Yield Gap Atlas. Weather data was retrieved from CHIRPS (precipitation) and WorldClim (temperature and radiation).

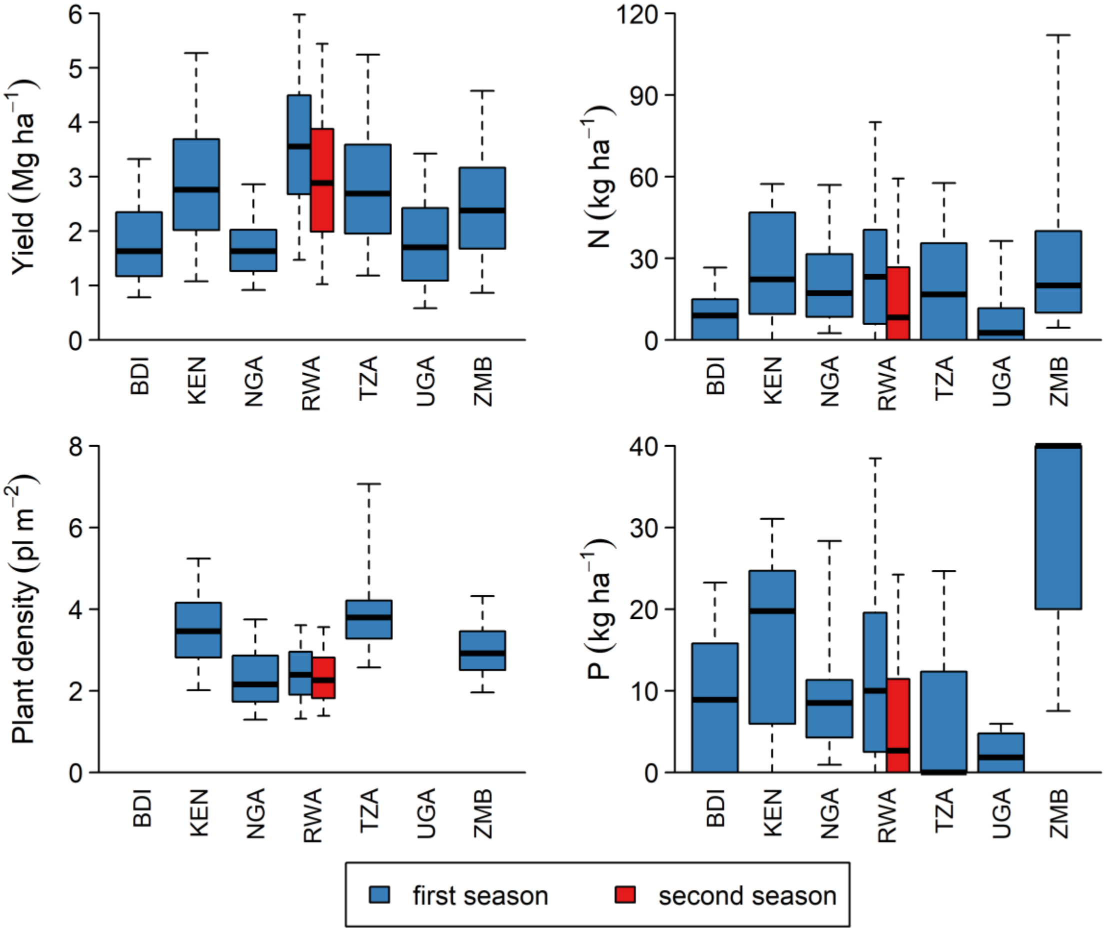

Fig. S2. Maize yield and main crop management practices in each country. Box-plots of yield and crop management variables in 14,773 maize fields included in the field-level farmer database in seven countries of Sub-Saharan Africa: Burundi (BDI), Kenya (KEN), Nigeria (NGA), Rwanda (RWA), Tanzania (TZA), Uganda (UGA), and Zambia (ZMB). Rwanda data was divided by season: first season (“season A”) and second season (“season B”). Nitrogen (N) and phosphorous (P) rates were calculated from inorganic fertilizers without considering compost. Plant density data was not available in BDI and UGA. Boxplots whiskers span from quantiles 0.1 to 0.9, boxes from quantile 0.25 to 0.75, and the center line indicates the median of each variable. The geolocation of all observations is shown in **Fig. 2.**

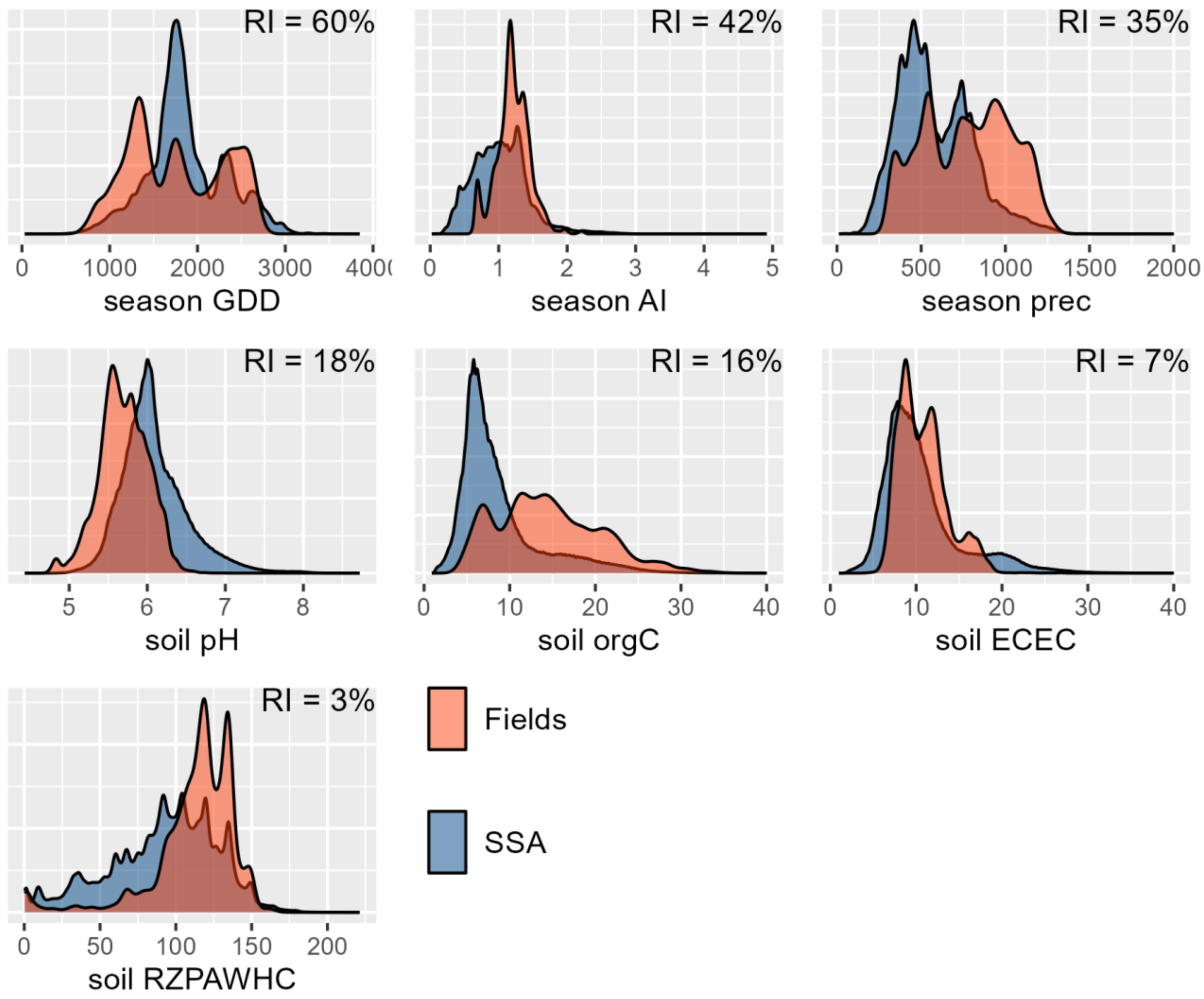

Fig. S3. Biophysical conditions in maize fields and Sub-Saharan Africa. Comparison of the distribution of selected climatic conditions during the maize growing season and selected soil properties in the 14,773 maize fields included in the field-level farmer database (“Fields”) vs. those across Sub-Saharan Africa maize area (SSA). The relative importance (RI) of each environmental variable for maize yield according to the Gradient Boosting Machine model is shown. Variable description, resolution, and data sources are provided in Table S2. Source data are provided as a Source Data file.

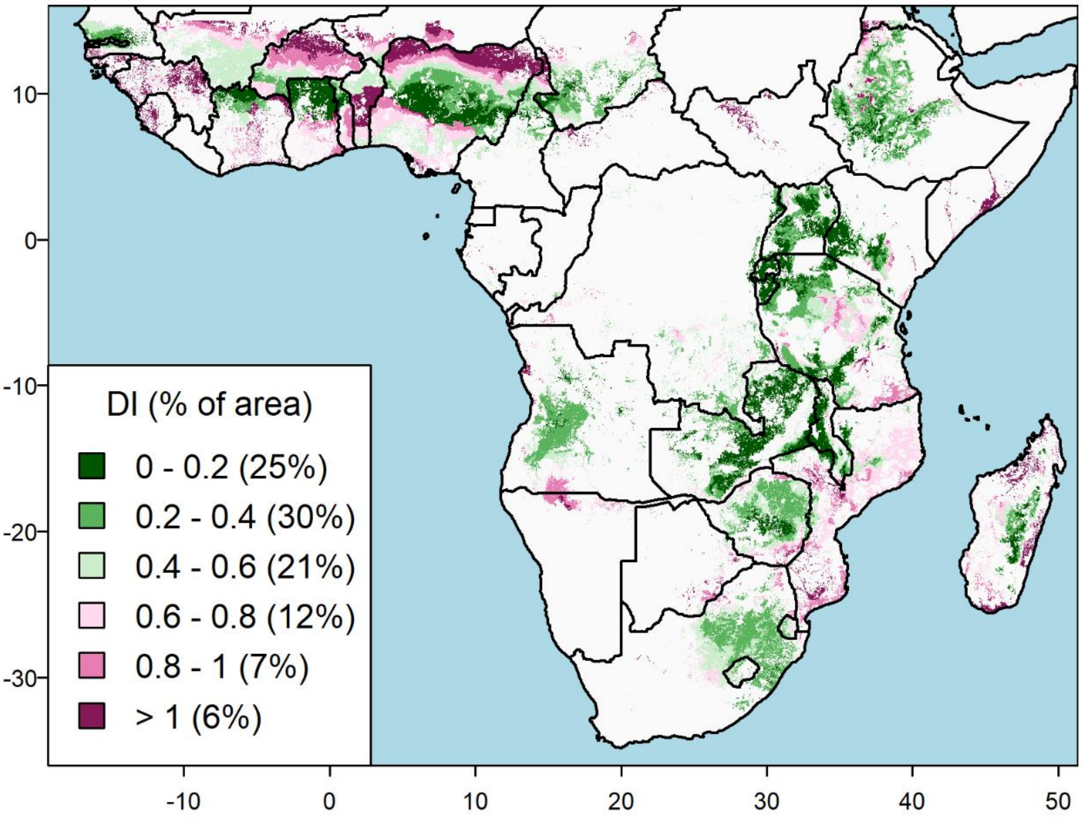

Fig. S4. Dissimilarity between maize fields and Sub-Saharan Africa harvested area. The Dissimilarity index (DI) was computed following Meyer and Pebesma[1]. DI represents the distance in the environmental space to the most similar field in the database. Included environmental variables are listed in **Table S2**. DI was computed by weighting environmental variables according to their relative importance in the Gradient Boosting Machine model. DI was standardized by dividing it by the average distance in the environmental space among all fields in the database. The percentage of maize area in Sub-Saharan Africa (SSA) that falls in each DI range is shown in the map legend. Source data are provided as a Source Data file.

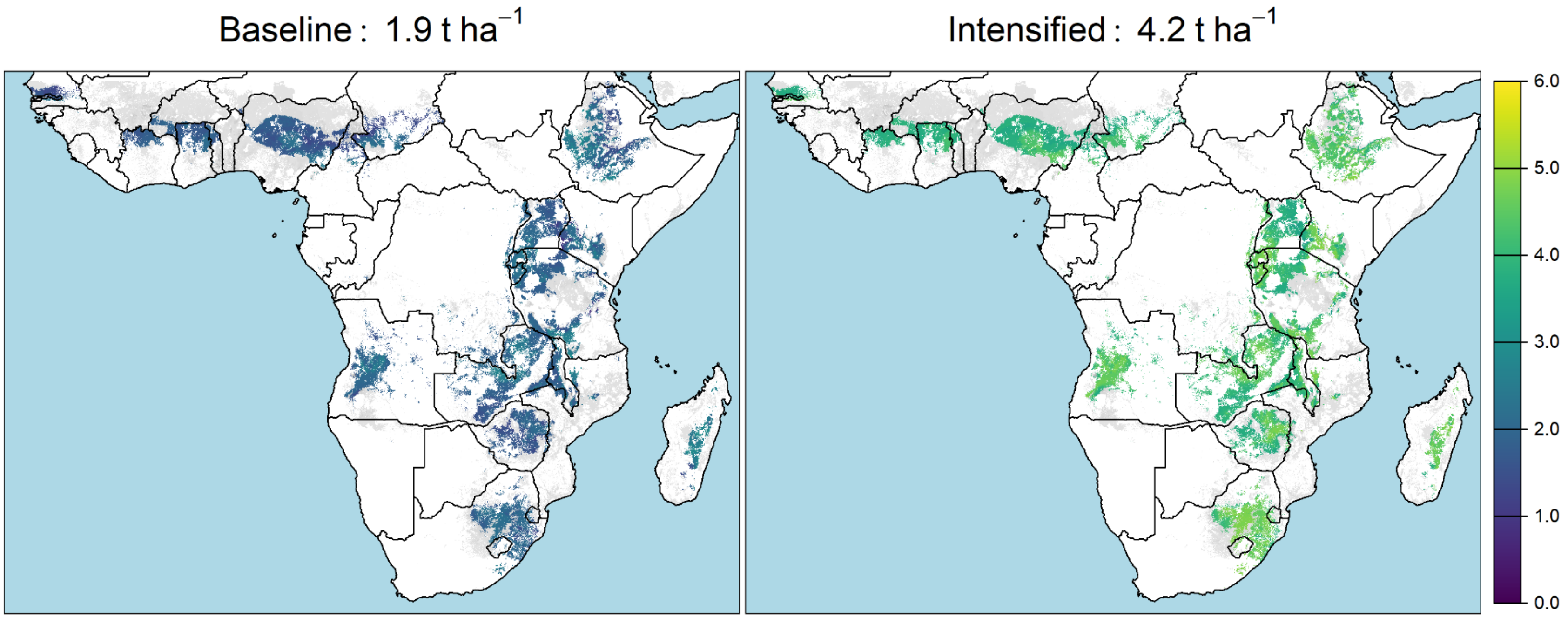

Fig. S5. Maize yield in Sub-Saharan Africa for two suites of management practices. Baseline management (left) includes open pollination varieties, no fertilizer application, no pest control, low plant densities (2 plants m^{-2}), and late sowing dates (10 days later than CZ average). Intensified management (right) includes use of hybrids, in-hole application of 60 kg $ha^{-1}$of N and 40 kg $ha^{-1}$of P, compost use, weeding, pesticide application, high plant density (5 plants m^{-2}), and early sowing date (15 days earlier than CZ average). Yields were predicted with a Gradient Boosting Machine model in maize areas in SSA with similar biophysical properties to those in the field-level farmers data used to train the model (i.e., areas with a dissimilarity index < 40% as shown in Fig. S4, which represents 55% of SSA maize area). Gray areas were excluded from model prediction due to their high dissimilarity with the biophysical conditions of the farmer fields included in our data. Source data are provided as a Source Data file.

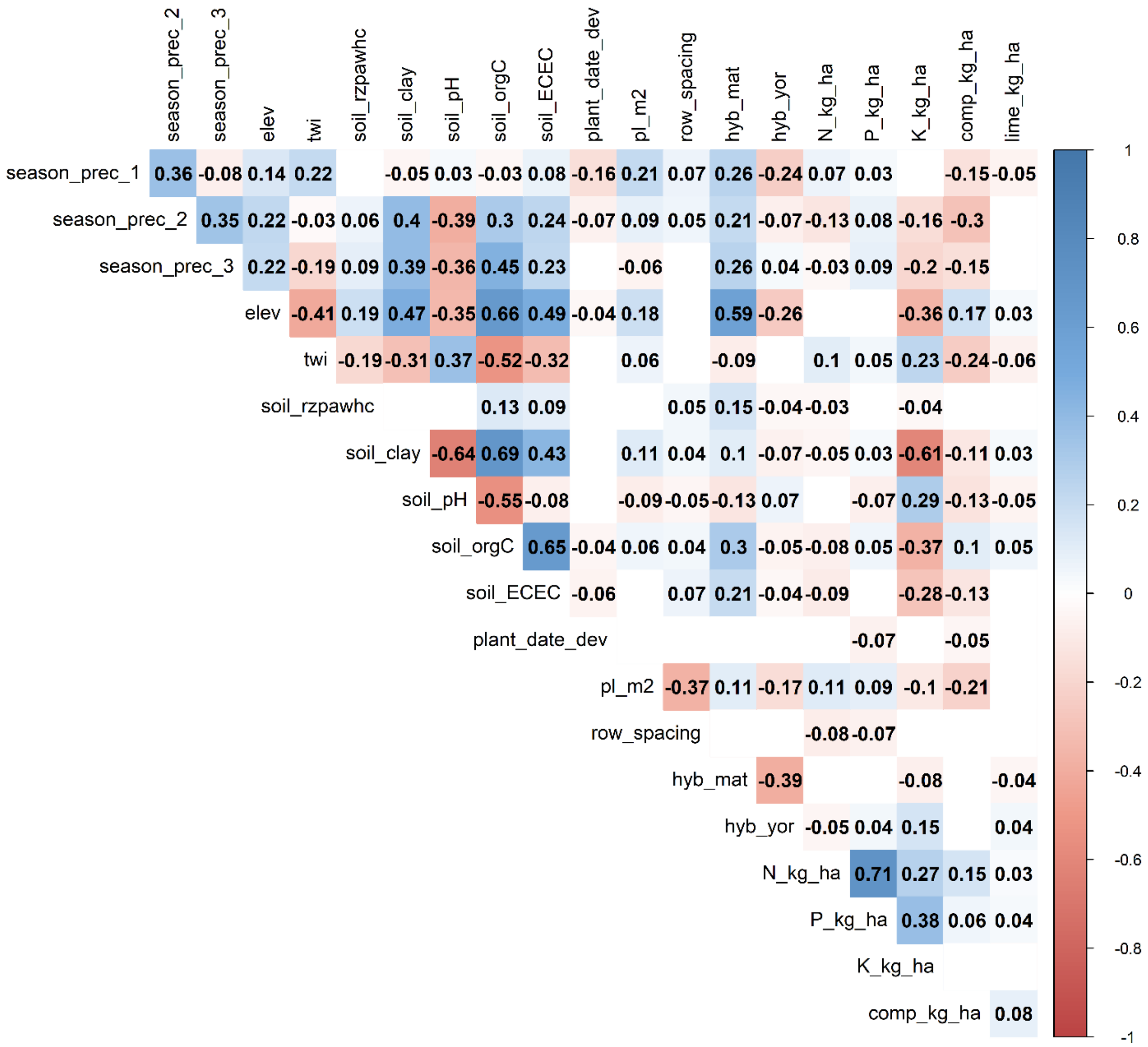

Fig. S6. Correlation coefficients of continuous variables. Pearson's correlation coefficients between all pairs of continuous predictor variables used for the conditional inference tree analysis. Empty squares indicate non-significant correlations (Pearson's correlation test P-value >0.01). Variable descriptions are shown in Table S1 Table S2. Source data are provided as a Source Data file.

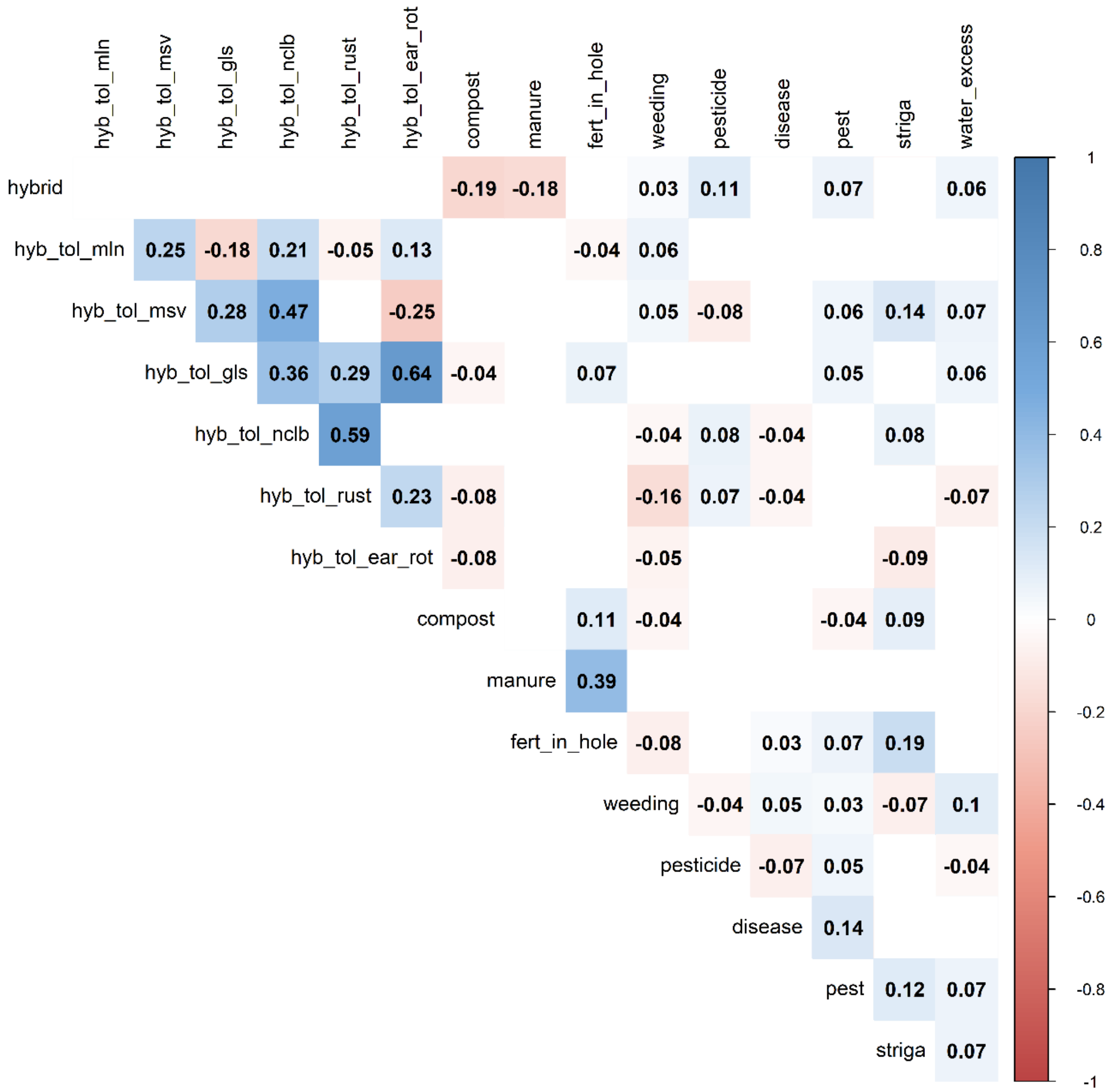

Fig. S7. Correlation coefficients of categorical variables. Phi correlation coefficients between all pairs of binary predictor variables used for the conditional inference tree analysis. Empty squares indicate non-significant correlations (Chi-square test P-value >0.01). Variable descriptions are shown in Table S1 Table S2. Source data are provided as a Source Data file.

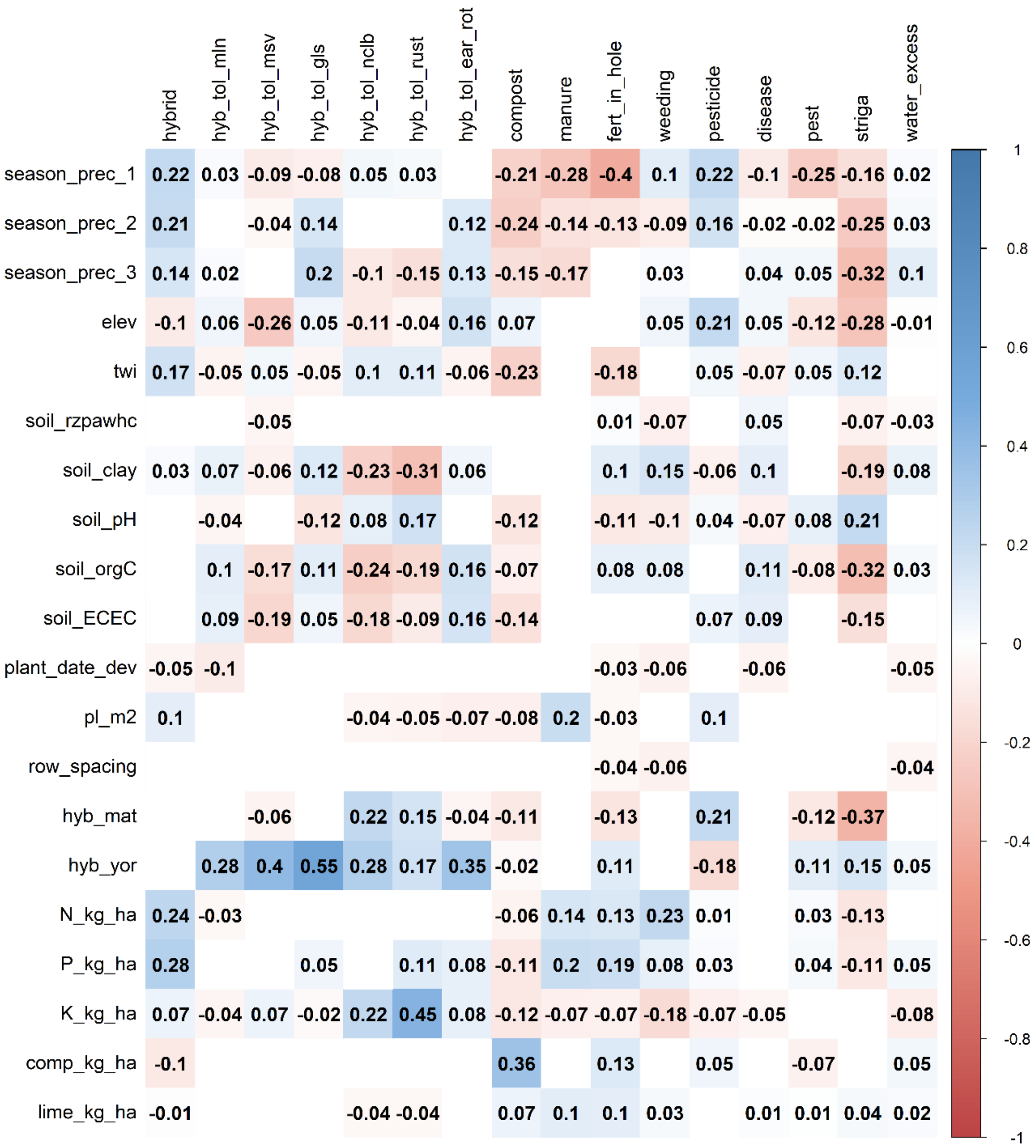

Fig. S8. Correlation coefficients between continuous and categorical variables. Point-biserial correlation coefficients between all combinations of binary (columns) and continuous (rows) predictor variables used for the conditional inference tree analysis. Empty squares indicate non-significant correlations (Kruskal-Wallis' test P-value >0.01). Variable descriptions are shown in Table S1 Table S2. Source data are provided as a Source Data file.

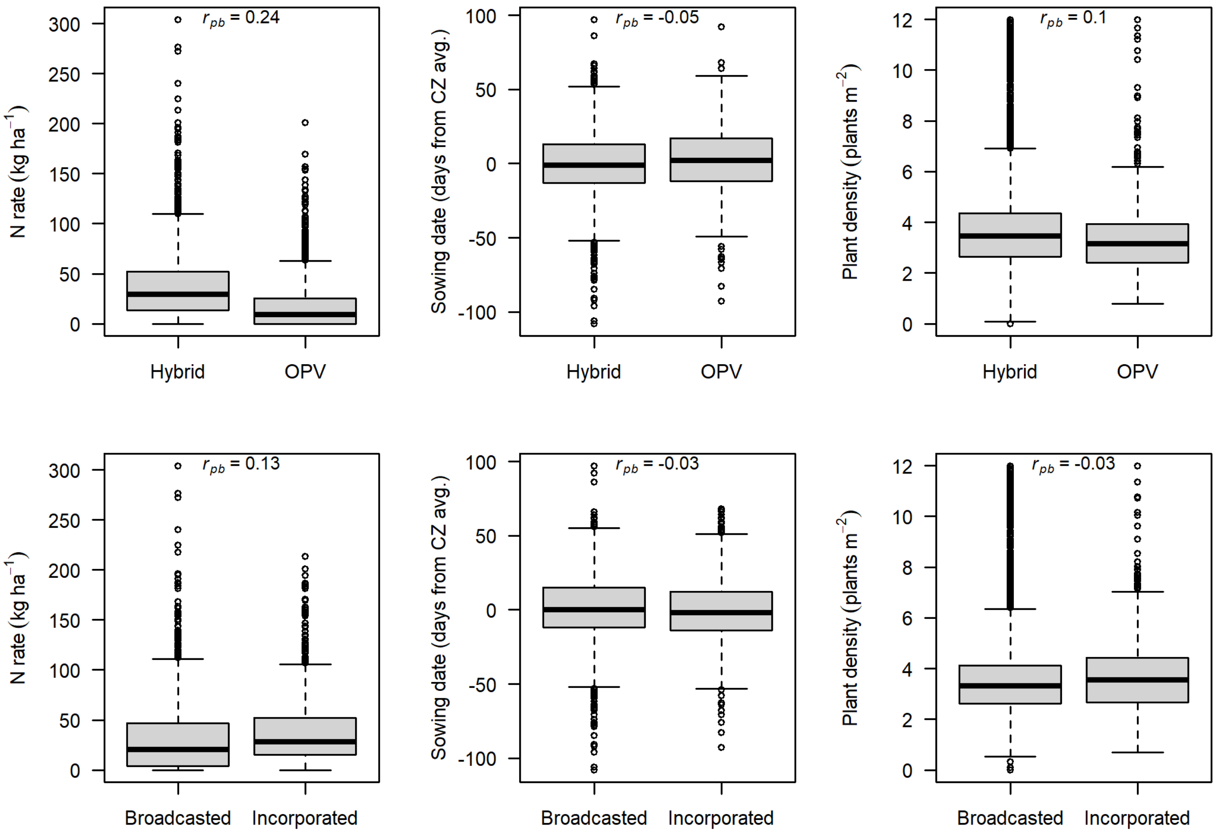

Fig. S9. Association between key continuous and binary management variables. Binary management variables include hybrid seed use (hybrid or open pollination varieties, OPV) and fertilization application method (broadcasted or incorporated). Key continuous management variables include N fertilization rate, sowing date deviation, and plant density. The central line of each boxplot indicates the median, the box encompasses the interquartile range, and the whiskers extend up to 1.5 times the interquartile range while more extreme values are depicted individually as outliers. The point-biserial correlation coefficient (r_{pb}) for each pair of variables is shown. Source data are provided as a Source Data file.

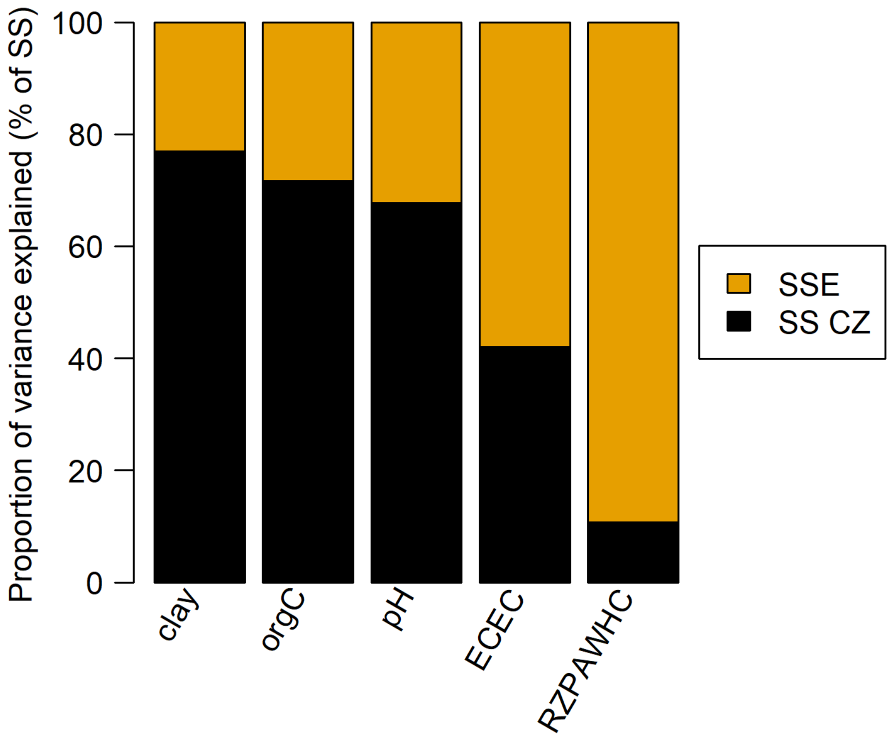

Fig. S10. Proportion of soil variance captured by climate zones. For each soil property, the proportion of variance explained was calculated with an analysis of variance (ANOVA) with soil property as dependent variable and climate zone (CZ) as independent variable. SS CZ is the percentage of the total sum of squares captured by differences in CZ (i.e., inter-CZ variation). SSE is the residual sum of squares (i.e., intra-CZ variation). Soil properties descriptions, resolutions, and data sources are provided in Table S2. Source data are provided as a Source Data file.

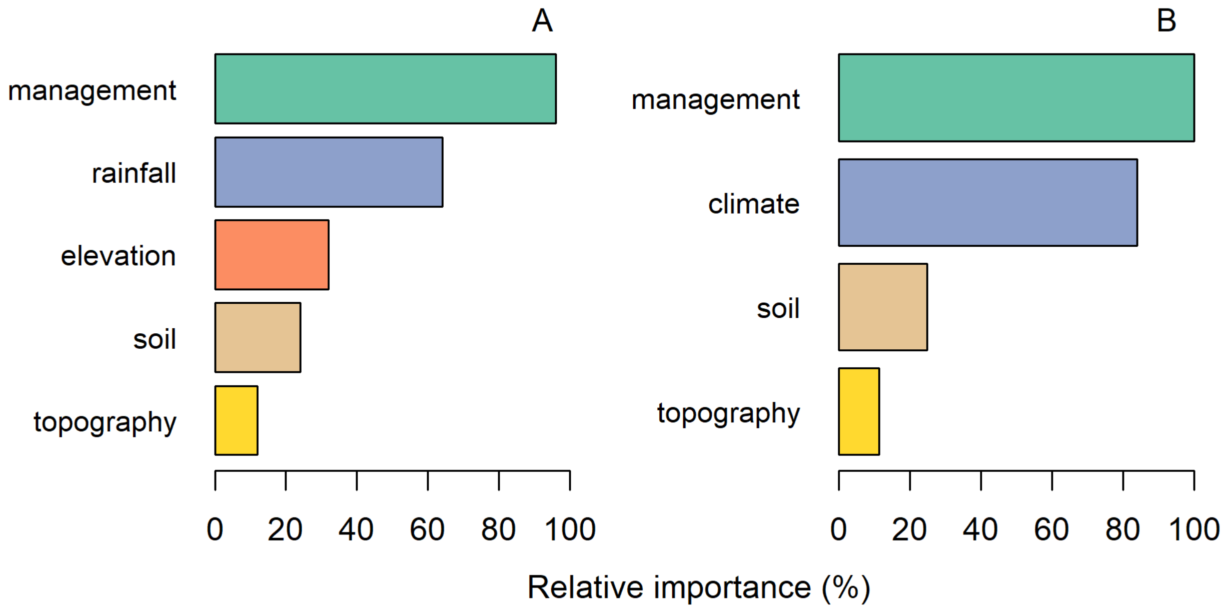

Fig. S11. Relative importance of factors explaining variation in maize yields. (A) Relative importance was calculated as the proportion of climate zones where an environmental variable or agronomic practice had a significant effect on maize yield, as identified in the conditional inference tree analysis (CIT, Fig. S12). (B) Relative importance as retrieved from the Gradient Boosting Machine (GBM) model, based on the relative number of times a variable was used in the trees. Elevation was only considered in the CIT to account for fine spatial variation in temperature within CZs. In B, the climate category includes the climatic variables used to define the CZs (long-term average growing degree days, aridity index, and temperature seasonality) and seasonal rainfall, all of which were considered for the GMB model. Source data are provided as a Source Data file.

Fig. S12. Conditional inference trees for each climate zone in the analysis(CZ #1 to #25). Management and environmental variables included in the conditional inference tree analysis are shown in Table S1 and Table S2. The country, climate zone properties, season, and years with data for each cluster are shown in the top left corner of each panel. GDD is the growing degree days range, and AI is the aridity index range (x 10,000) (see van Wart et al., 2013). Boxplots show the yield distribution observed at each environmental and crop management combination. In each boxplot, the center line indicates the median yield, the box encompasses the interquartile range, and the whiskers span from 0.1 to 0.9 quantiles of the yield distribution. The number of observations (n) and average maize yield are also shown. n and the p-value of the variable's effect on yield are also indicated at each node (data partition). Model fit statistics (R^2 and root mean square error, RMSE) are shown in the top right corner of each panel. Source data are provided as a Source Data file. Source Code used for the analysis is provided in Supplementary Code.

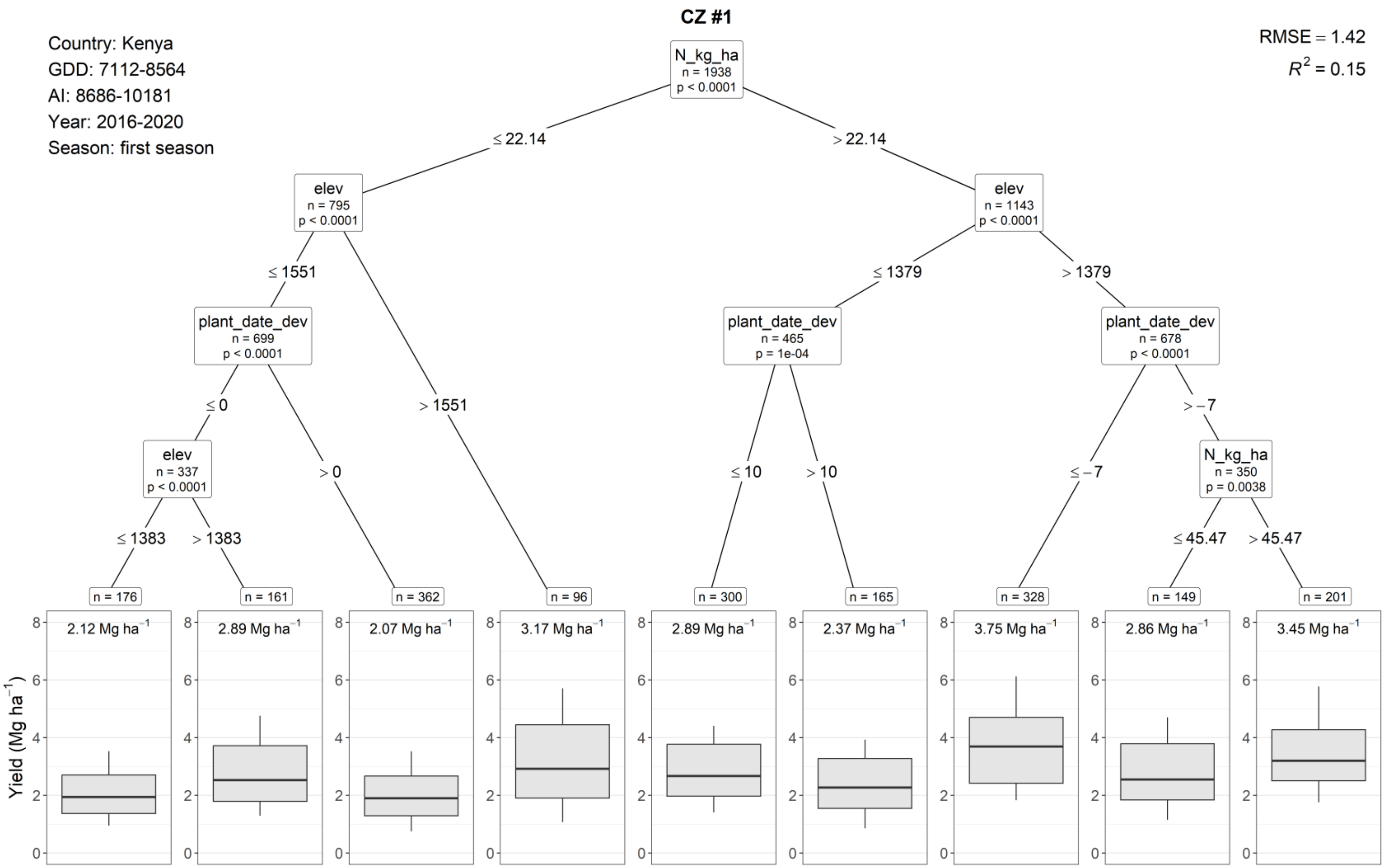
CZ #1
Country: Kenya
GDD: 7112-8564
AI: 8686-10181
Year: 2016-2020
Season: first season
RMSE = 1.42
R^2 = 0.15
N_kg_ha
n = 1938
p < 0.0001
≤ 22.14
> 22.14
elev
n = 795
p < 0.0001
elev
n = 1143
p < 0.0001
≤ 1551
> 1551
≤ 1379
> 1379
plant_date_dev
n = 699
p < 0.0001
plant_date_dev
n = 465
p = 1e-04
plant_date_dev
n = 678
p < 0.0001
≤ 0
> 0
≤ 10
> 10
≤ −7
> −7
elev
n = 337
p < 0.0001
N_kg_ha
n = 350
p = 0.0038
≤ 1383
> 1383
≤ 45.47
> 45.47
n = 176
n = 161
n = 362
n = 96
n = 300
n = 165
n = 328
n = 149
n = 201
2.12 Mg ha^{-1}
2.89 Mg ha^{-1}
2.07 Mg ha^{-1}
3.17 Mg ha^{-1}
2.89 Mg ha^{-1}
2.37 Mg ha^{-1}
3.75 Mg ha^{-1}
2.86 Mg ha^{-1}
3.45 Mg ha^{-1}
Yield (Mg ha^{-1})
0
2
4
6
8

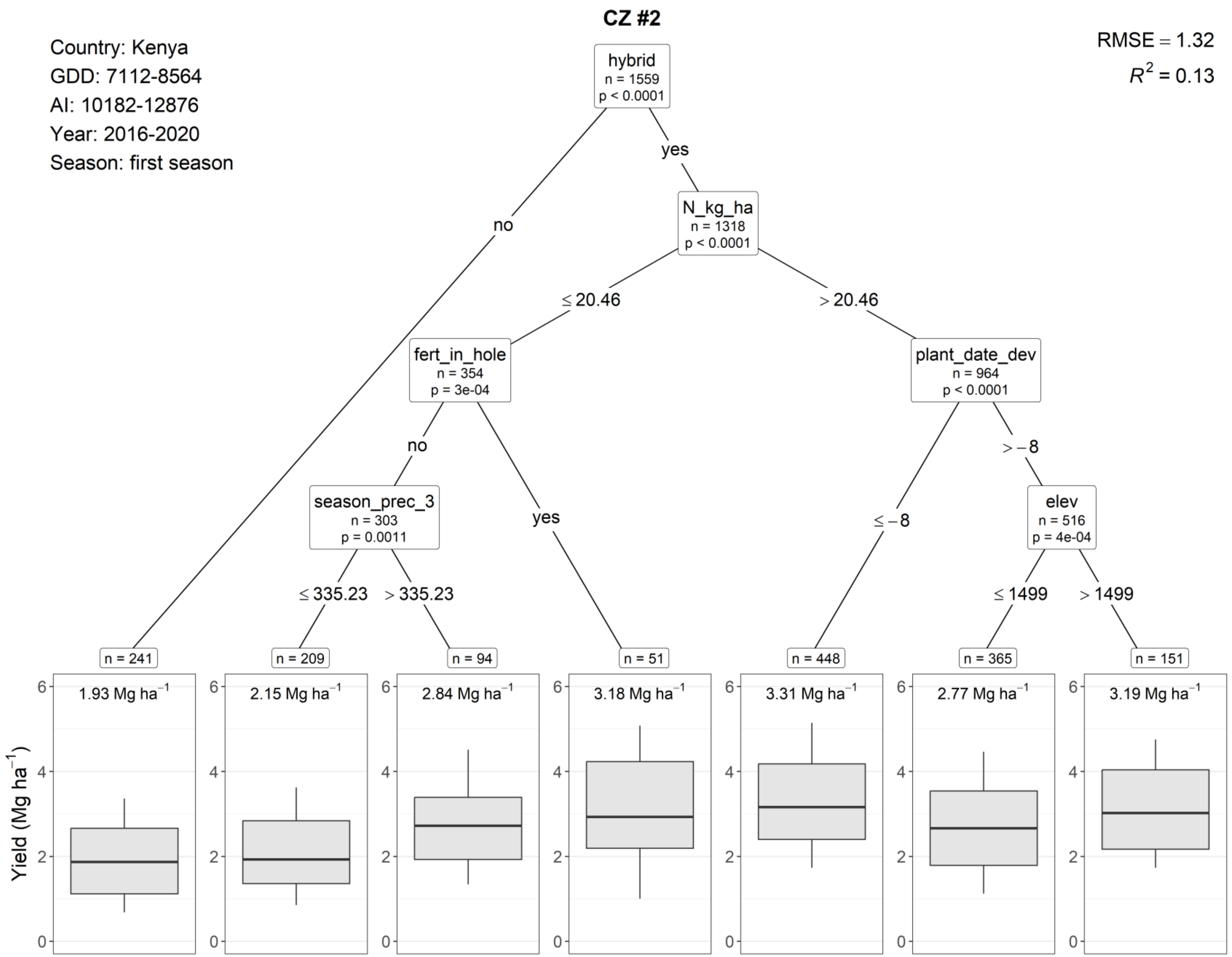

CZ #2
Country: Kenya
GDD: 7112-8564
AI: 10182-12876
Year: 2016-2020
Season: first season
RMSE = 1.32
R^2 = 0.13
hybrid
n = 1559
p < 0.0001
no
yes
N_kg_ha
n = 1318
p < 0.0001
≤ 20.46
> 20.46
fert_in_hole
n = 354
p = 3e-04
plant_date_dev
n = 964
p < 0.0001
no
yes
> −8
≤ −8
season_prec_3
n = 303
p = 0.0011
elev
n = 516
p = 4e-04
≤ 335.23
> 335.23
≤ 1499
> 1499
n = 241
n = 209
n = 94
n = 51
n = 448
n = 365
n = 151
1.93 Mg ha^{-1}
2.15 Mg ha^{-1}
2.84 Mg ha^{-1}
3.18 Mg ha^{-1}
3.31 Mg ha^{-1}
2.77 Mg ha^{-1}
3.19 Mg ha^{-1}
Yield (Mg ha^{-1})
0
2
4
6

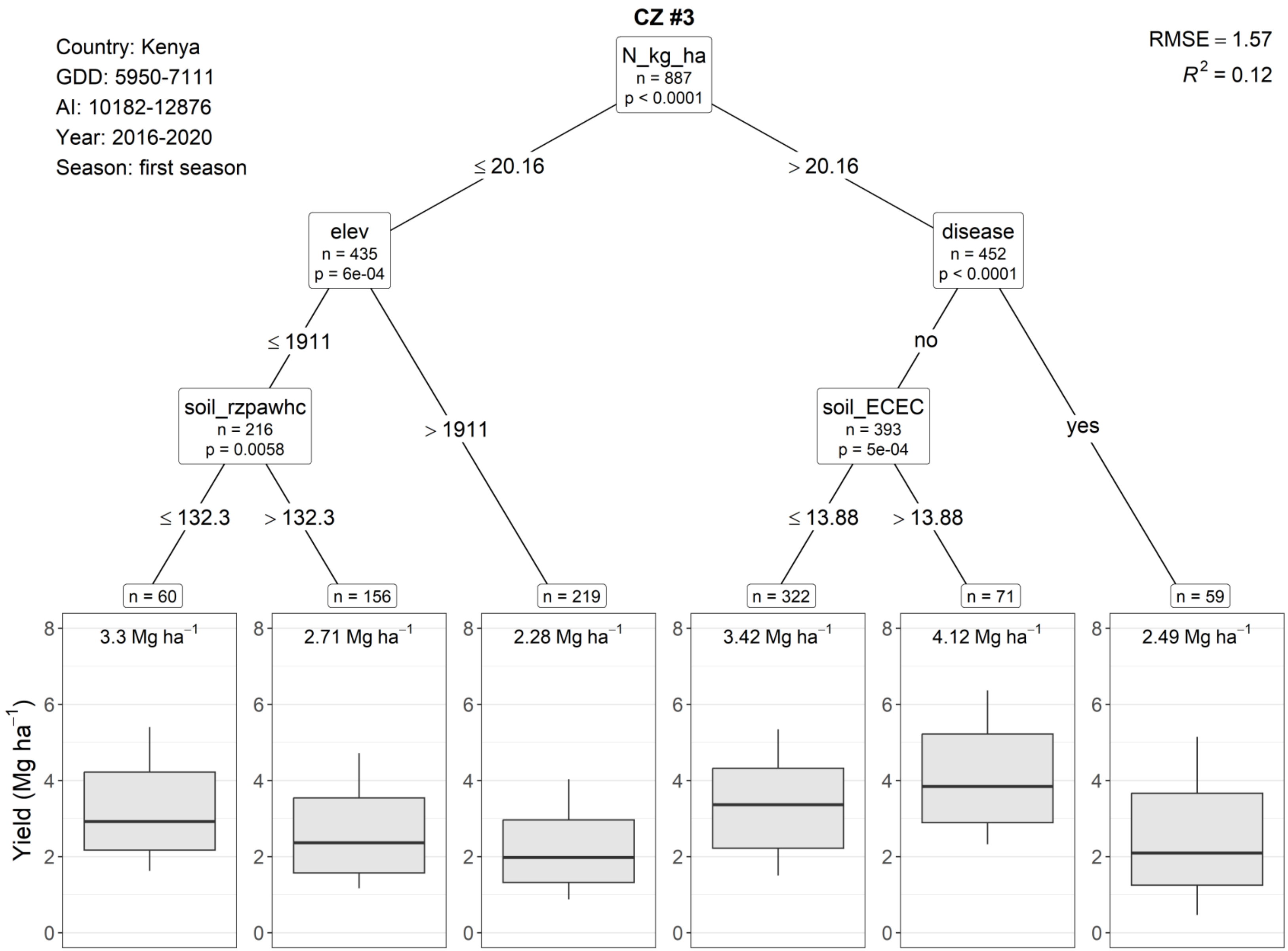
CZ #3
Country: Kenya
GDD: 5950-7111
AI: 10182-12876
Year: 2016-2020
Season: first season
RMSE = 1.57
R2 = 0.12
N_kg_ha
n = 887
p < 0.0001
≤ 20.16
> 20.16
elev
n = 435
p = 6e-04
disease
n = 452
p < 0.0001
≤ 1911
> 1911
no
yes
soil_rzpawhc
n = 216
p = 0.0058
soil_ECEC
n = 393
p = 5e-04
≤ 132.3
> 132.3
≤ 13.88
> 13.88
n = 60
n = 156
n = 219
n = 322
n = 71
n = 59
3.3 Mg ha−1
2.71 Mg ha−1
2.28 Mg ha−1
3.42 Mg ha−1
4.12 Mg ha−1
2.49 Mg ha−1
Yield (Mg ha−1)
0
2
4
6
8

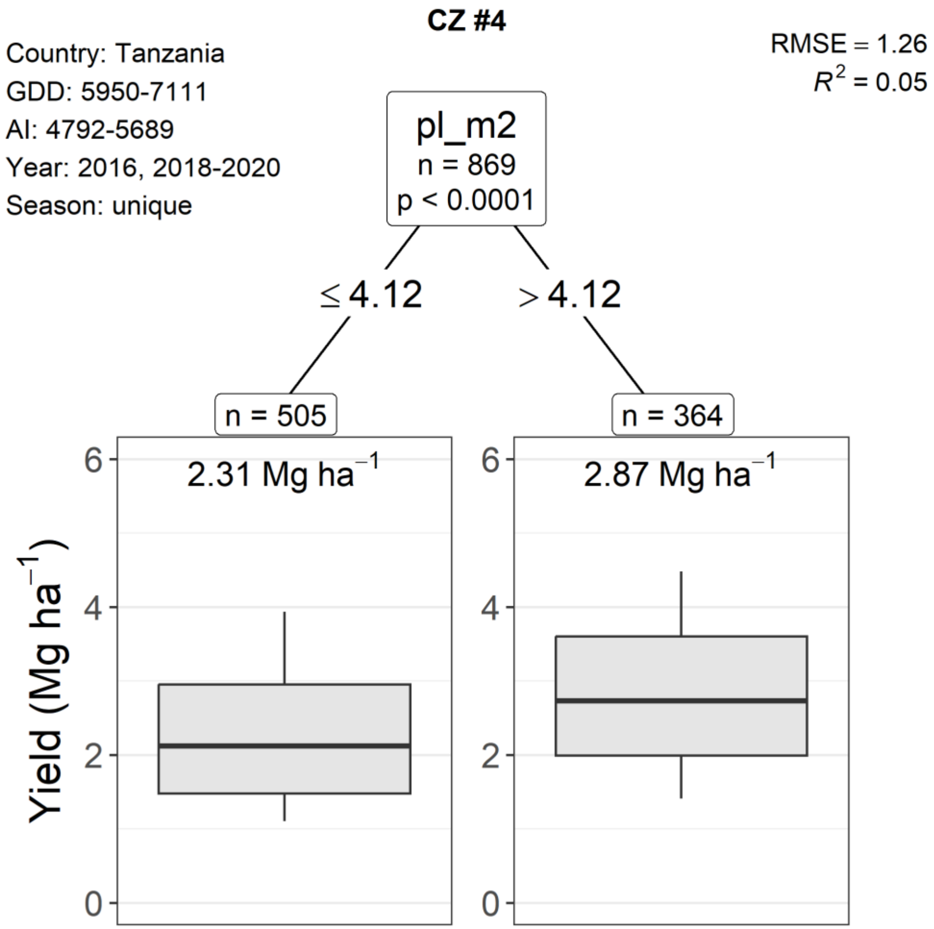
CZ #4
Country: Tanzania
GDD: 5950-7111
AI: 4792-5689
Year: 2016, 2018-2020
Season: unique
RMSE = 1.26
R^2 = 0.05
pl_m2
n = 869
p < 0.0001
≤ 4.12
> 4.12
n = 505
n = 364
2.31 Mg ha^{-1}
2.87 Mg ha^{-1}
Yield (Mg ha^{-1})
0
2
4
6

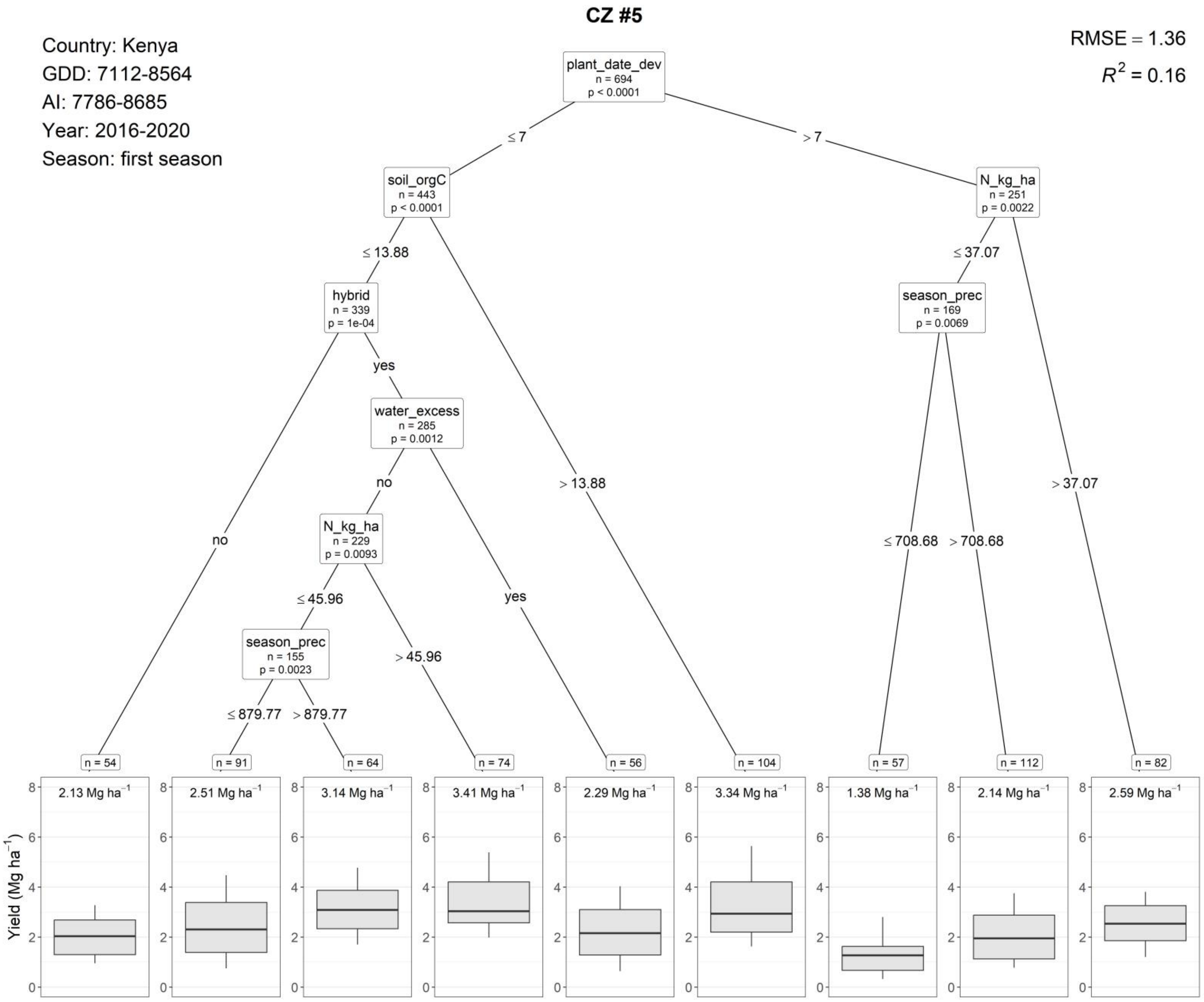
CZ #5
Country: Kenya
GDD: 7112-8564
AI: 7786-8685
Year: 2016-2020
Season: first season
RMSE = 1.36
R^2 = 0.16
plant_date_dev
n = 694
p < 0.0001
≤ 7
> 7
soil_orgC
n = 443
p < 0.0001
N_kg_ha
n = 251
p = 0.0022
≤ 13.88
> 13.88
hybrid
n = 339
p = 1e-04
no
yes
water_excess
n = 285
p = 0.0012
no
yes
N_kg_ha
n = 229
p = 0.0093
≤ 45.96
> 45.96
season_prec
n = 155
p = 0.0023
≤ 879.77
> 879.77
≤ 37.07
> 37.07
season_prec
n = 169
p = 0.0069
≤ 708.68
> 708.68
n = 54
n = 91
n = 64
n = 74
n = 56
n = 104
n = 57
n = 112
n = 82
2.13 Mg ha^{-1}
2.51 Mg ha^{-1}
3.14 Mg ha^{-1}
3.41 Mg ha^{-1}
2.29 Mg ha^{-1}
3.34 Mg ha^{-1}
1.38 Mg ha^{-1}
2.14 Mg ha^{-1}
2.59 Mg ha^{-1}
Yield (Mg ha^{-1})
0
2
4
6
8

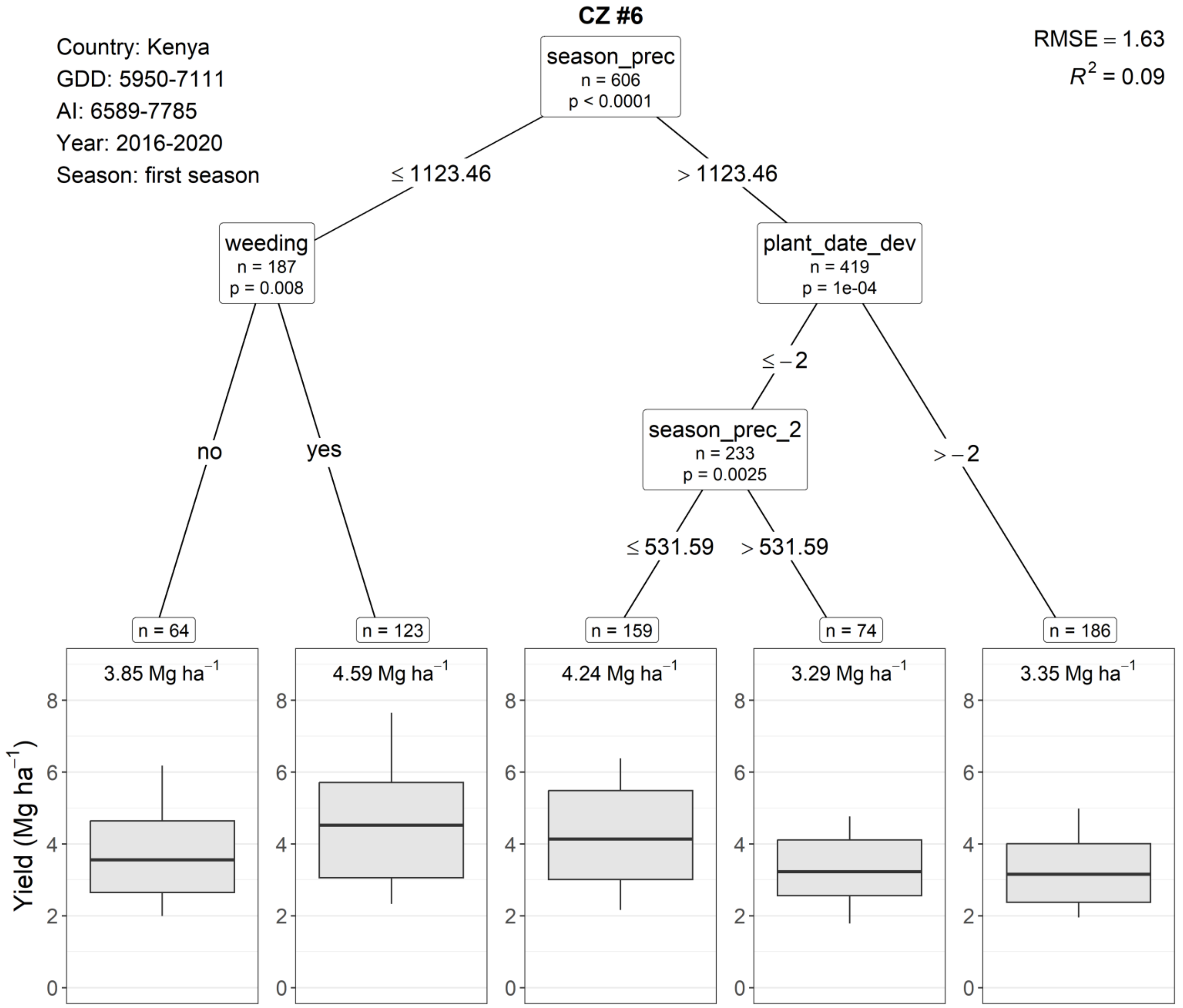
CZ #6
Country: Kenya
GDD: 5950-7111
AI: 6589-7785
Year: 2016-2020
Season: first season
RMSE = 1.63
R^2 = 0.09
season_prec
n = 606
p < 0.0001
≤ 1123.46
> 1123.46
weeding
n = 187
p = 0.008
plant_date_dev
n = 419
p = 1e-04
no
yes
≤ −2
> −2
season_prec_2
n = 233
p = 0.0025
≤ 531.59
> 531.59
n = 64
n = 123
n = 159
n = 74
n = 186
3.85 Mg ha^{-1}
4.59 Mg ha^{-1}
4.24 Mg ha^{-1}
3.29 Mg ha^{-1}
3.35 Mg ha^{-1}
Yield (Mg ha^{-1})
0
2
4
6
8

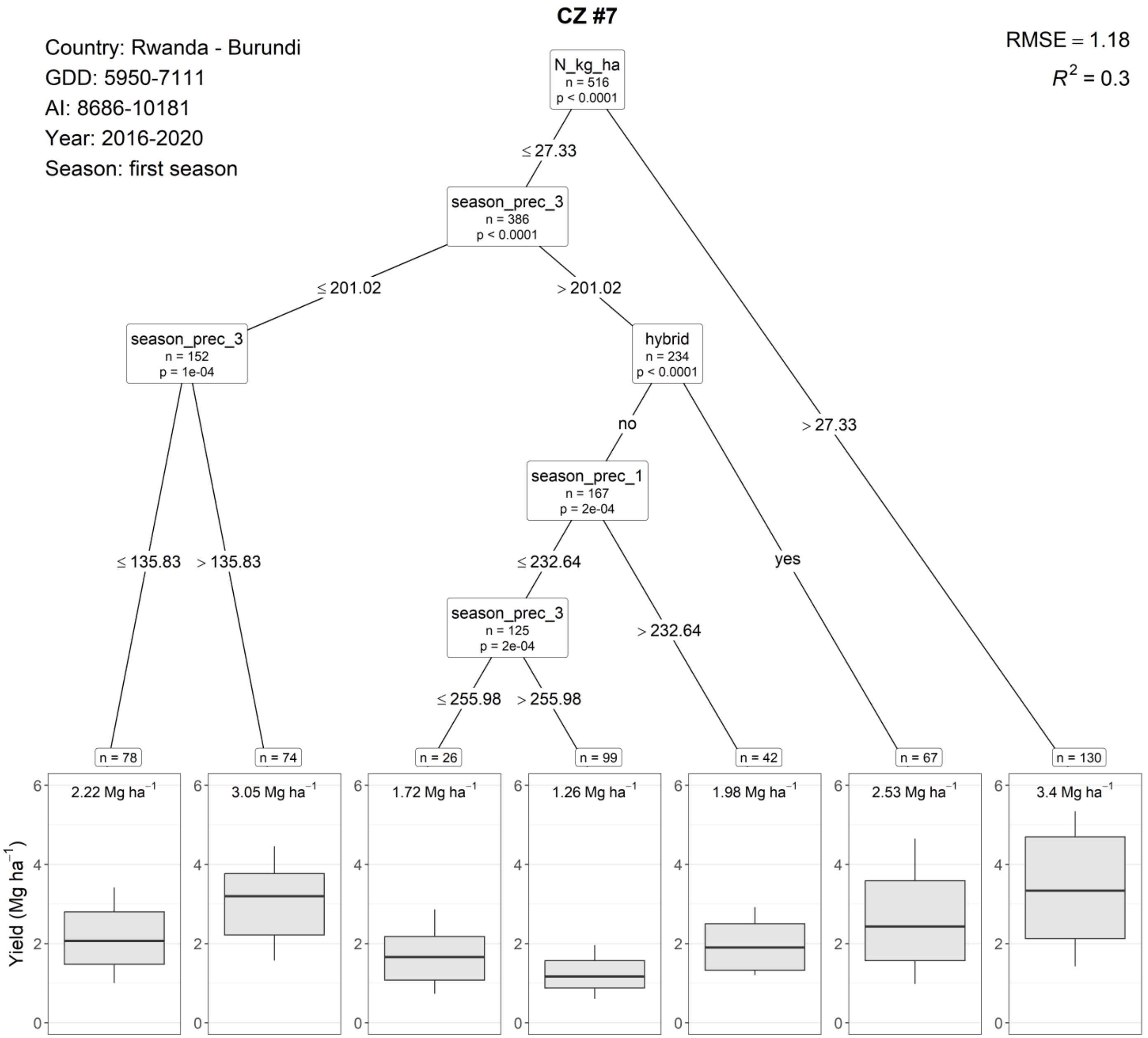
CZ #7
Country: Rwanda - Burundi
GDD: 5950-7111
AI: 8686-10181
Year: 2016-2020
Season: first season
RMSE = 1.18
R^2 = 0.3
N_kg_ha
n = 516
p < 0.0001
≤ 27.33
> 27.33
season_prec_3
n = 386
p < 0.0001
≤ 201.02
> 201.02
season_prec_3
n = 152
p = 1e-04
≤ 135.83
> 135.83
hybrid
n = 234
p < 0.0001
no
yes
season_prec_1
n = 167
p = 2e-04
≤ 232.64
> 232.64
season_prec_3
n = 125
p = 2e-04
≤ 255.98
> 255.98
n = 78
n = 74
n = 26
n = 99
n = 42
n = 67
n = 130
2.22 Mg ha^{-1}
3.05 Mg ha^{-1}
1.72 Mg ha^{-1}
1.26 Mg ha^{-1}
1.98 Mg ha^{-1}
2.53 Mg ha^{-1}
3.4 Mg ha^{-1}
Yield (Mg ha^{-1})
0
2
4
6

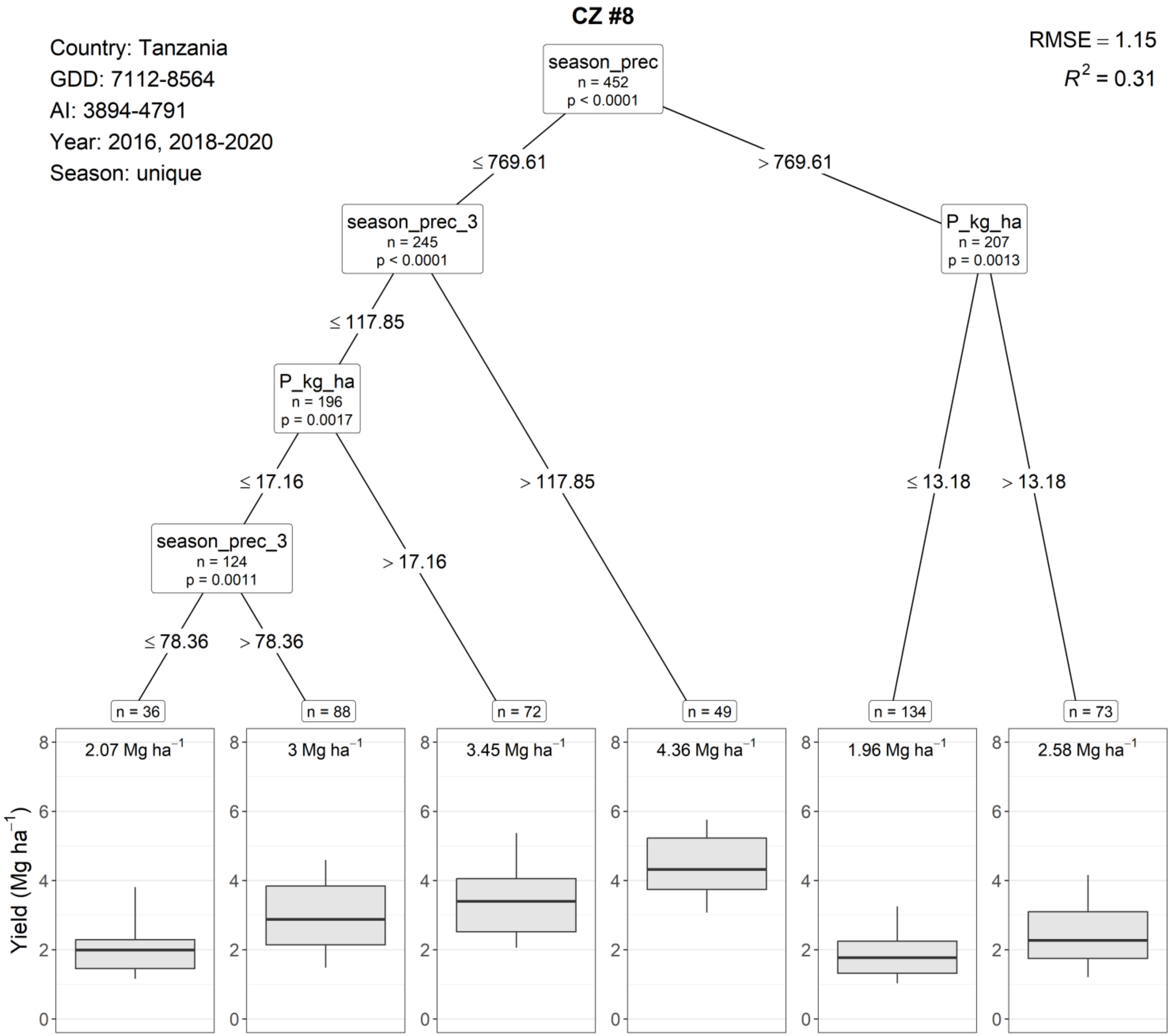
CZ #8
Country: Tanzania
GDD: 7112-8564
AI: 3894-4791
Year: 2016, 2018-2020
Season: unique
RMSE = 1.15
R2 = 0.31
season_prec
n = 452
p < 0.0001
≤ 769.61
> 769.61
season_prec_3
n = 245
p < 0.0001
P_kg_ha
n = 207
p = 0.0013
≤ 117.85
> 117.85
P_kg_ha
n = 196
p = 0.0017
≤ 17.16
> 17.16
≤ 13.18
> 13.18
season_prec_3
n = 124
p = 0.0011
≤ 78.36
> 78.36
n = 36
n = 88
n = 72
n = 49
n = 134
n = 73
2.07 Mg ha-1
3 Mg ha-1
3.45 Mg ha-1
4.36 Mg ha-1
1.96 Mg ha-1
2.58 Mg ha-1
Yield (Mg ha-1)
0
2
4
6
8

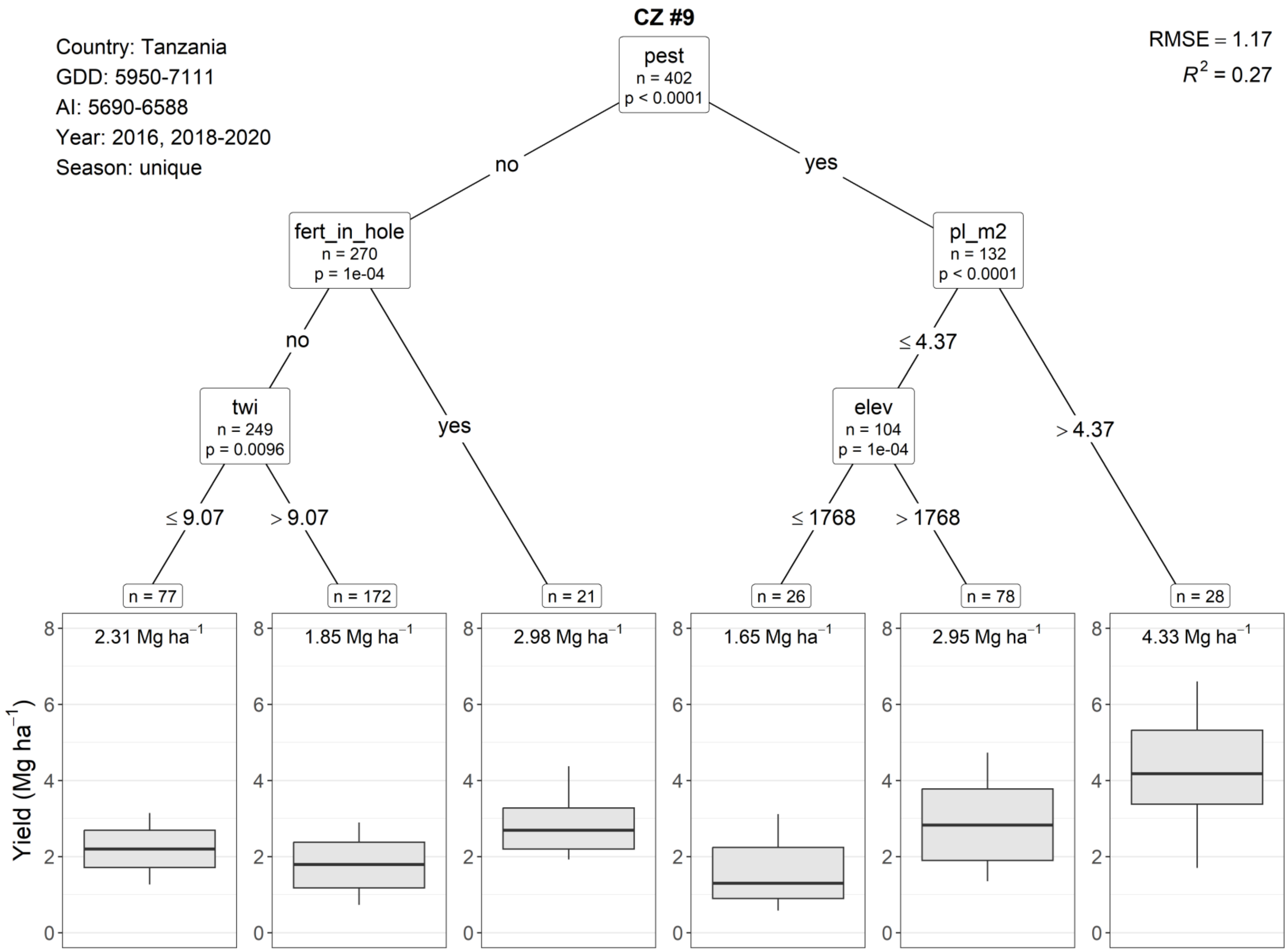
CZ #9
Country: Tanzania
GDD: 5950-7111
AI: 5690-6588
Year: 2016, 2018-2020
Season: unique
RMSE = 1.17
R^2 = 0.27
pest
n = 402
p < 0.0001
no
yes
fert_in_hole
n = 270
p = 1e-04
pl_m2
n = 132
p < 0.0001
no
yes
≤ 4.37
> 4.37
twi
n = 249
p = 0.0096
elev
n = 104
p = 1e-04
≤ 9.07
> 9.07
≤ 1768
> 1768
n = 77
n = 172
n = 21
n = 26
n = 78
n = 28
2.31 Mg ha^{-1}
1.85 Mg ha^{-1}
2.98 Mg ha^{-1}
1.65 Mg ha^{-1}
2.95 Mg ha^{-1}
4.33 Mg ha^{-1}
Yield (Mg ha^{-1})
0
2
4
6
8

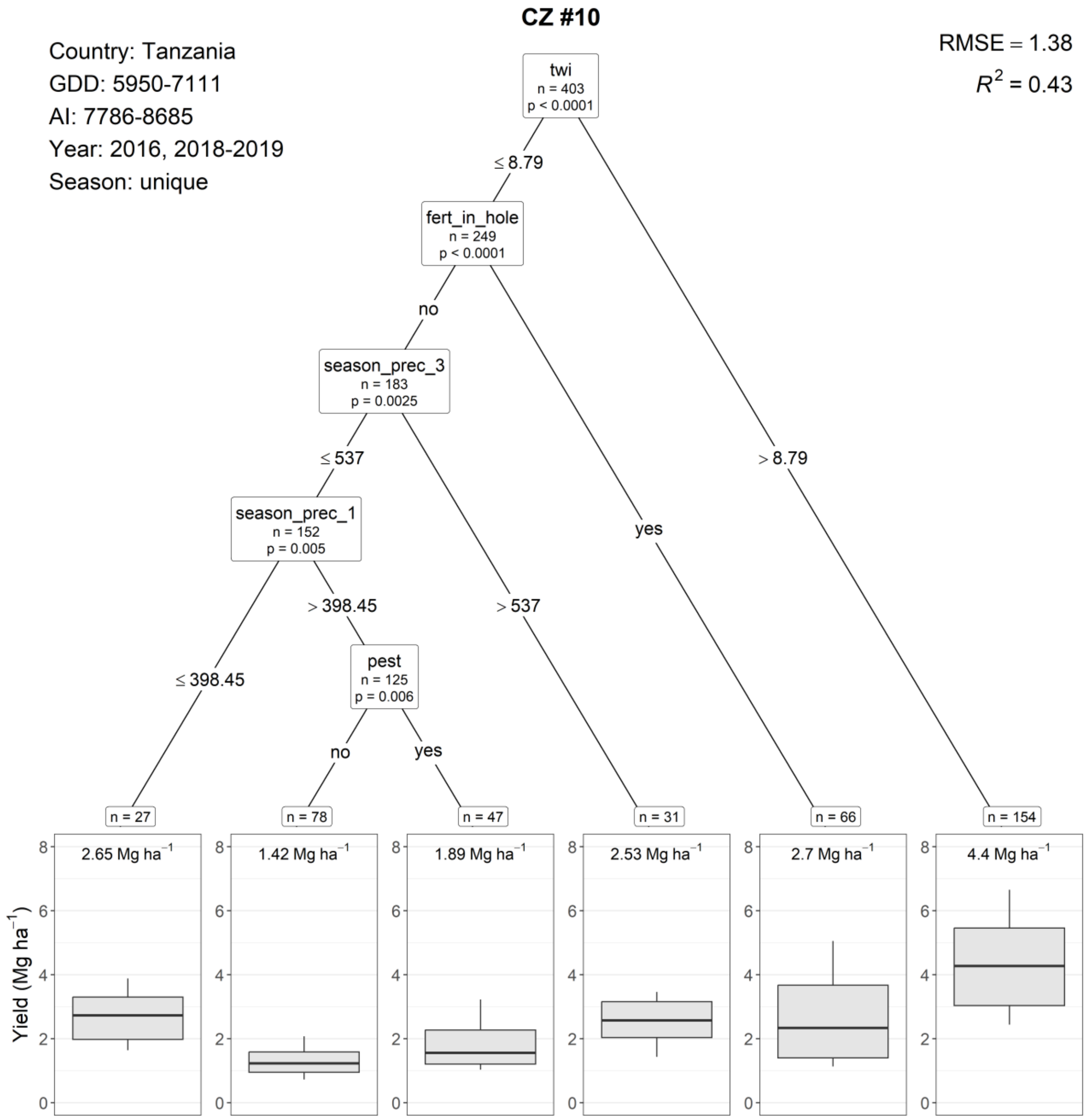

CZ #10
Country: Tanzania
GDD: 5950-7111
AI: 7786-8685
Year: 2016, 2018-2019
Season: unique
RMSE = 1.38
R^2 = 0.43
twi
n = 403
p < 0.0001
≤ 8.79
> 8.79
fert_in_hole
n = 249
p < 0.0001
no
yes
season_prec_3
n = 183
p = 0.0025
≤ 537
> 537
season_prec_1
n = 152
p = 0.005
≤ 398.45
> 398.45
pest
n = 125
p = 0.006
no
yes
n = 27
n = 78
n = 47
n = 31
n = 66
n = 154
2.65 Mg ha^{-1}
1.42 Mg ha^{-1}
1.89 Mg ha^{-1}
2.53 Mg ha^{-1}
2.7 Mg ha^{-1}
4.4 Mg ha^{-1}
Yield (Mg ha^{-1})
0
2
4
6
8

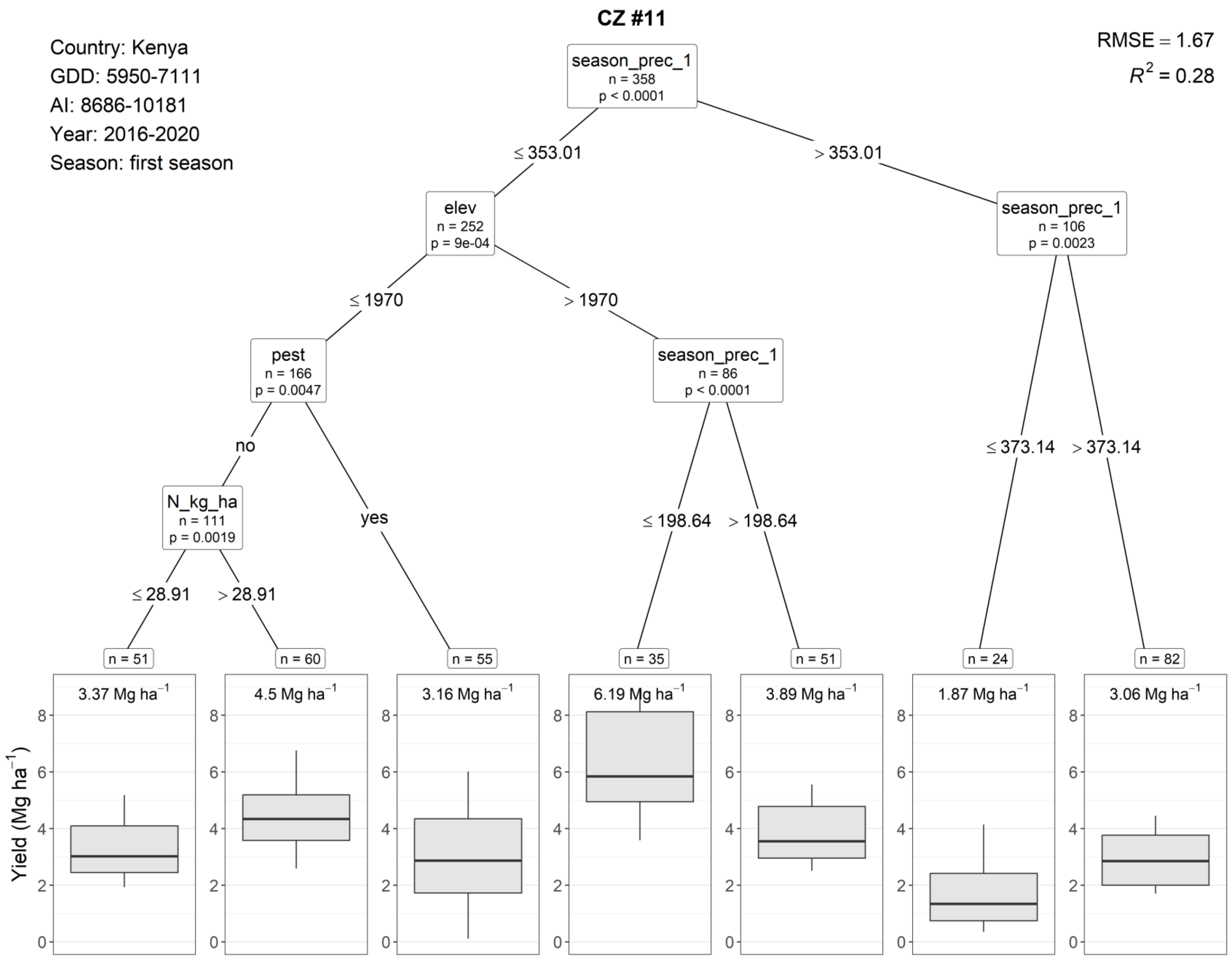

CZ #11
Country: Kenya
GDD: 5950-7111
AI: 8686-10181
Year: 2016-2020
Season: first season
RMSE = 1.67
R² = 0.28
season_prec_1
n = 358
p < 0.0001
≤ 353.01
> 353.01
elev
n = 252
p = 9e-04
season_prec_1
n = 106
p = 0.0023
≤ 1970
> 1970
pest
n = 166
p = 0.0047
season_prec_1
n = 86
p < 0.0001
≤ 373.14
> 373.14
no
yes
N_kg_ha
n = 111
p = 0.0019
≤ 198.64
> 198.64
≤ 28.91
> 28.91
n = 51
n = 60
n = 55
n = 35
n = 51
n = 24
n = 82
3.37 Mg ha⁻¹
4.5 Mg ha⁻¹
3.16 Mg ha⁻¹
6.19 Mg ha⁻¹
3.89 Mg ha⁻¹
1.87 Mg ha⁻¹
3.06 Mg ha⁻¹
Yield (Mg ha⁻¹)
0
2
4
6
8

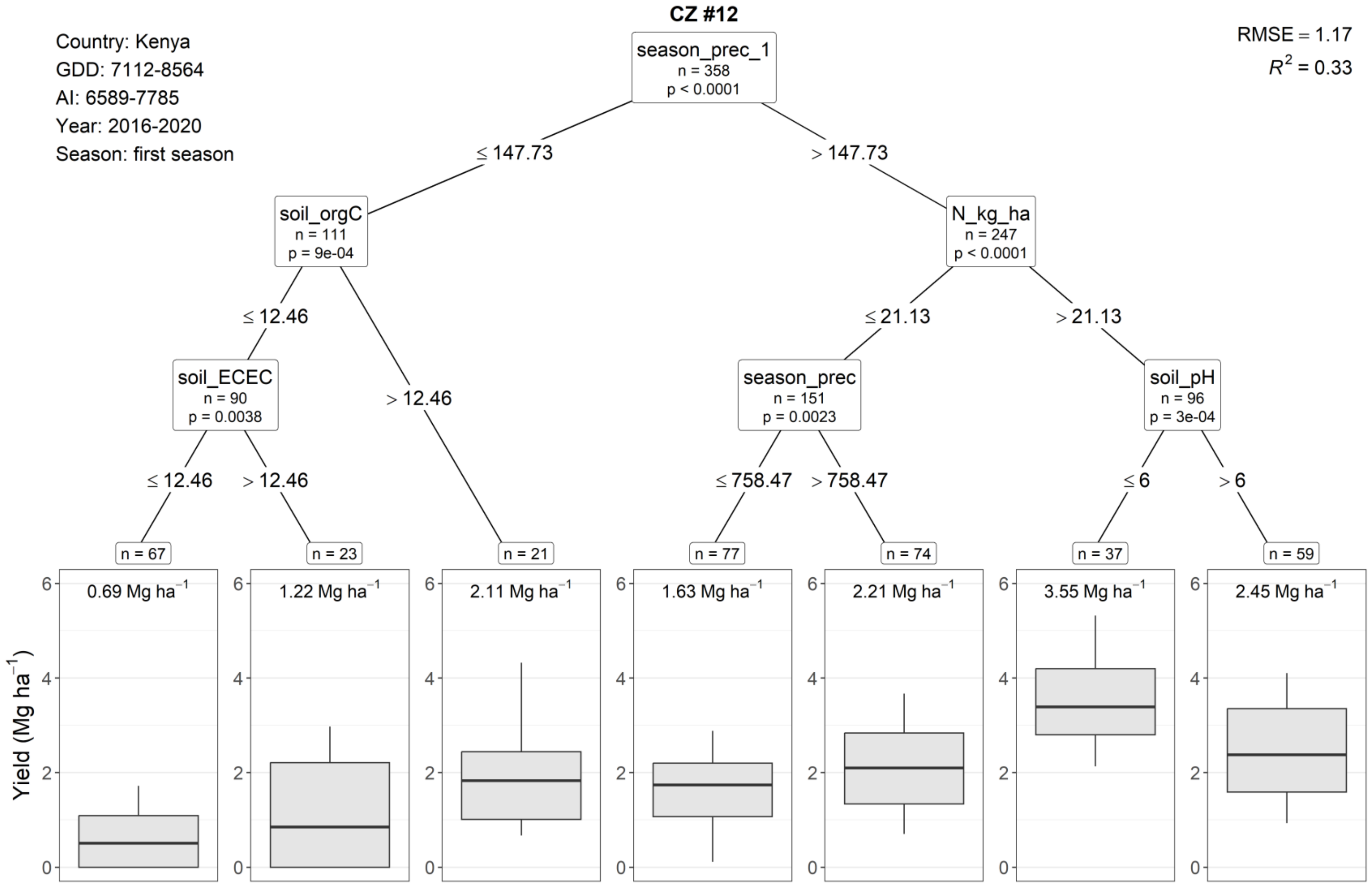
CZ #12
Country: Kenya
GDD: 7112-8564
AI: 6589-7785
Year: 2016-2020
Season: first season
RMSE = 1.17
R^2 = 0.33
season_prec_1
n = 358
p < 0.0001
≤ 147.73
> 147.73
soil_orgC
n = 111
p = 9e-04
N_kg_ha
n = 247
p < 0.0001
≤ 12.46
> 12.46
≤ 21.13
> 21.13
soil_ECEC
n = 90
p = 0.0038
season_prec
n = 151
p = 0.0023
soil_pH
n = 96
p = 3e-04
≤ 12.46
> 12.46
≤ 758.47
> 758.47
≤ 6
> 6
n = 67
n = 23
n = 21
n = 77
n = 74
n = 37
n = 59
0.69 Mg ha^{-1}
1.22 Mg ha^{-1}
2.11 Mg ha^{-1}
1.63 Mg ha^{-1}
2.21 Mg ha^{-1}
3.55 Mg ha^{-1}
2.45 Mg ha^{-1}
Yield (Mg ha^{-1})

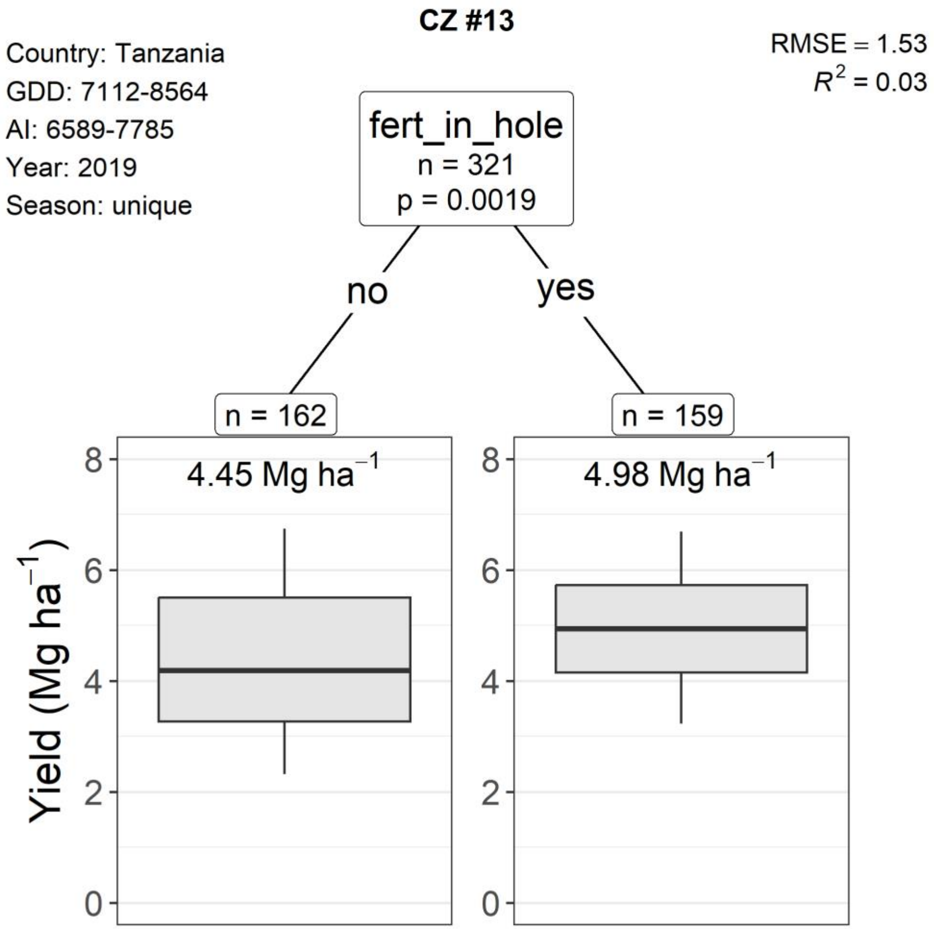
CZ #13
Country: Tanzania
GDD: 7112-8564
AI: 6589-7785
Year: 2019
Season: unique
RMSE = 1.53
R2 = 0.03
fert_in_hole
n = 321
p = 0.0019
no
yes
n = 162
n = 159
4.45 Mg ha−1
4.98 Mg ha−1
Yield (Mg ha−1)
8
6
4
2
0

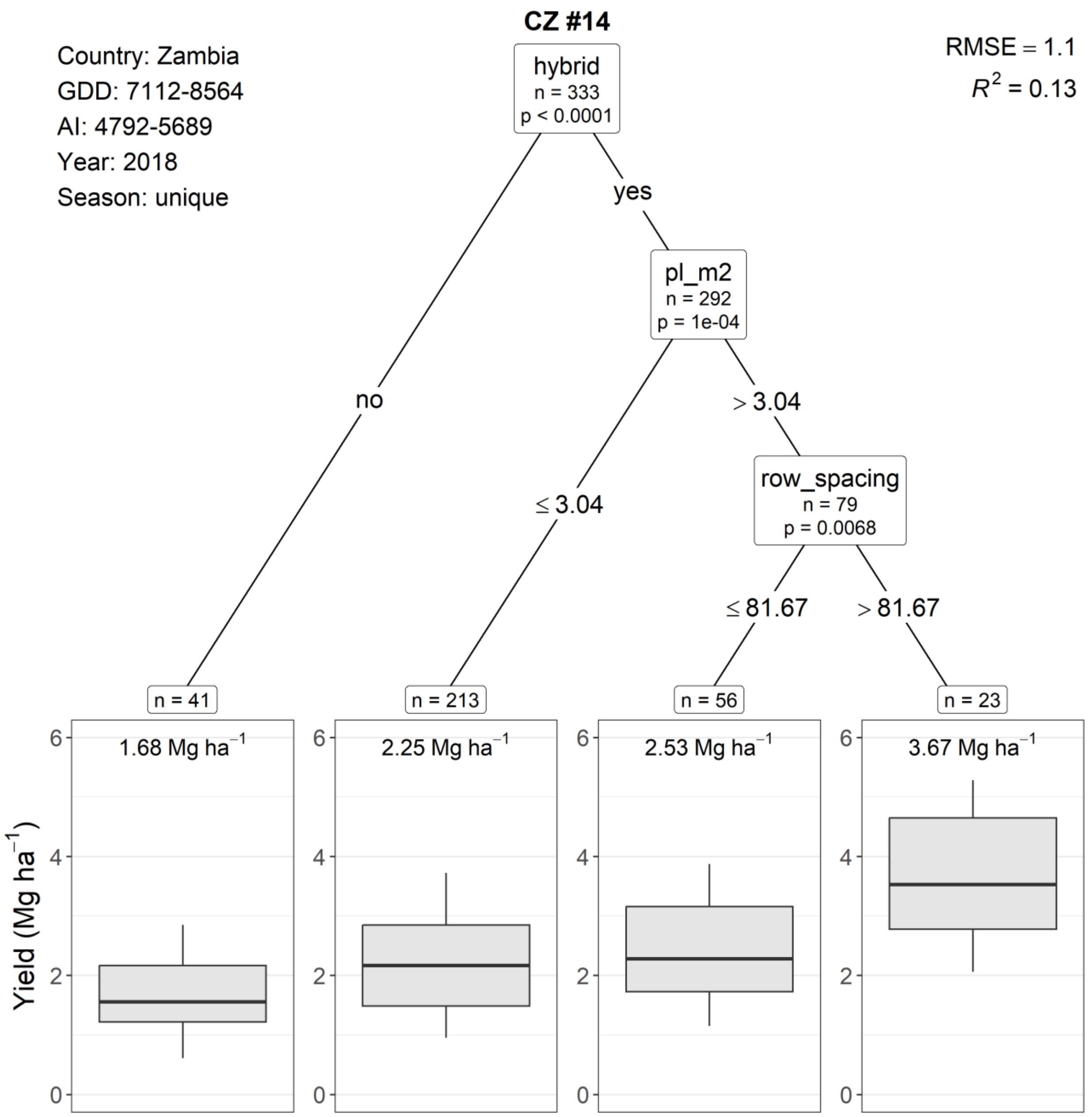
CZ #14
Country: Zambia
GDD: 7112-8564
AI: 4792-5689
Year: 2018
Season: unique
RMSE = 1.1
R^2 = 0.13
hybrid
n = 333
p < 0.0001
no
yes
pl_m2
n = 292
p = 1e-04
≤ 3.04
> 3.04
row_spacing
n = 79
p = 0.0068
≤ 81.67
> 81.67
n = 41
n = 213
n = 56
n = 23
1.68 Mg ha^{-1}
2.25 Mg ha^{-1}
2.53 Mg ha^{-1}
3.67 Mg ha^{-1}
Yield (Mg ha^{-1})
0
2
4
6

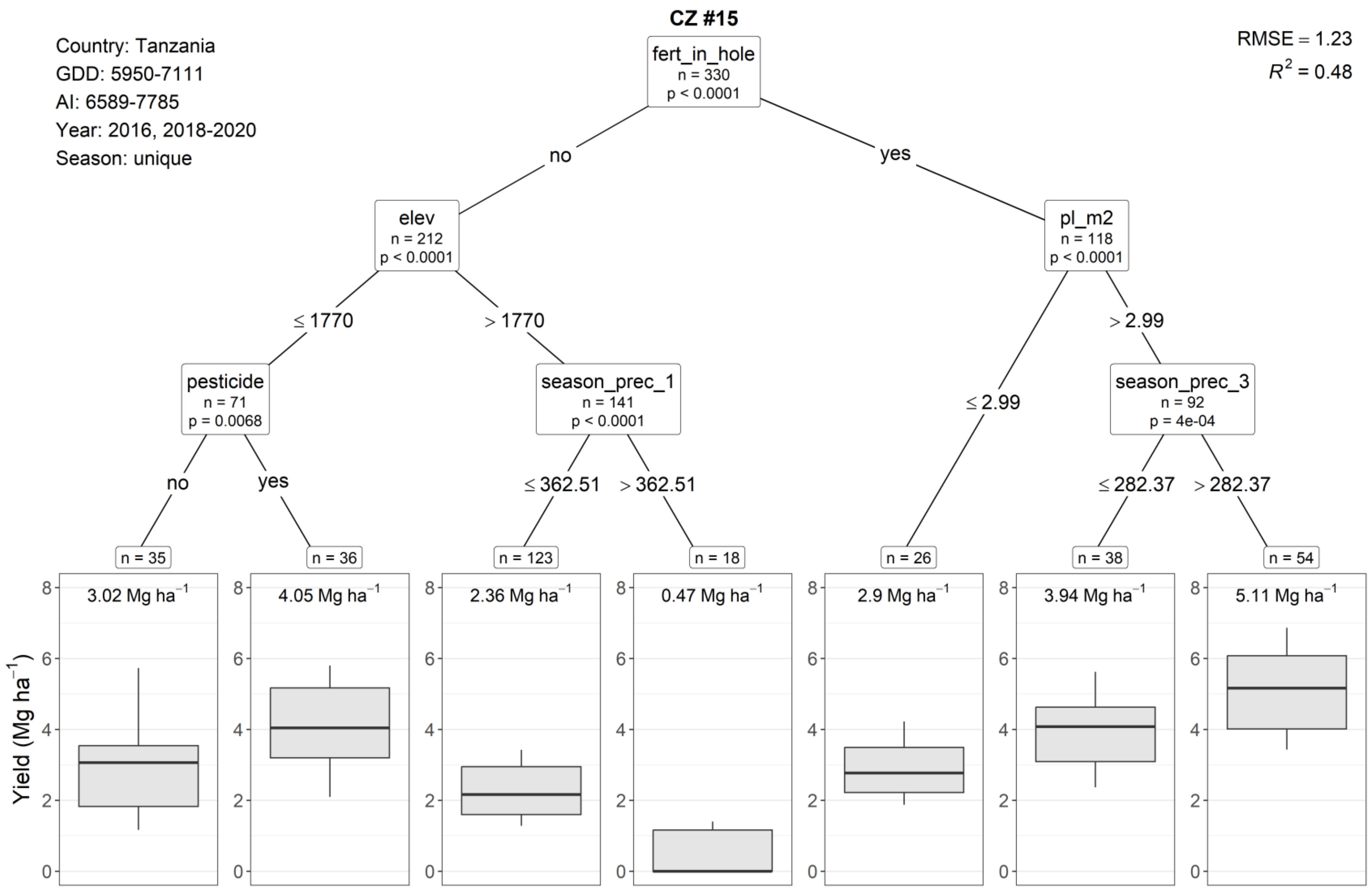
CZ #15
Country: Tanzania
GDD: 5950-7111
AI: 6589-7785
Year: 2016, 2018-2020
Season: unique
RMSE = 1.23
R^2 = 0.48
fert_in_hole
n = 330
p < 0.0001
no
yes
elev
n = 212
p < 0.0001
pl_m2
n = 118
p < 0.0001
≤ 1770
> 1770
> 2.99
≤ 2.99
pesticide
n = 71
p = 0.0068
season_prec_1
n = 141
p < 0.0001
season_prec_3
n = 92
p = 4e-04
no
yes
≤ 362.51
> 362.51
≤ 282.37
> 282.37
n = 35
n = 36
n = 123
n = 18
n = 26
n = 38
n = 54
3.02 Mg ha^{-1}
4.05 Mg ha^{-1}
2.36 Mg ha^{-1}
0.47 Mg ha^{-1}
2.9 Mg ha^{-1}
3.94 Mg ha^{-1}
5.11 Mg ha^{-1}
Yield (Mg ha^{-1})
0
2
4
6
8

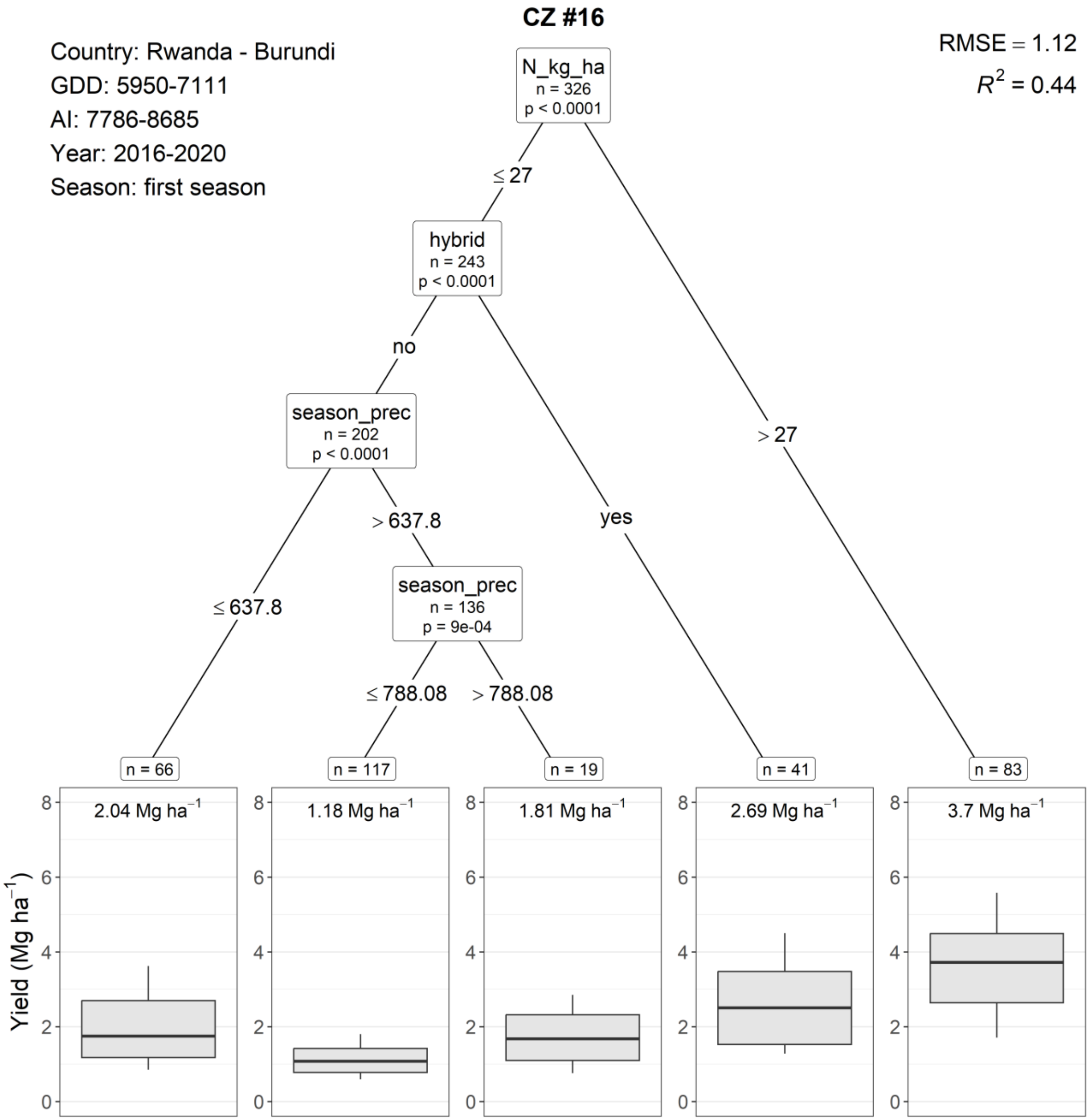
CZ #16
Country: Rwanda - Burundi
GDD: 5950-7111
AI: 7786-8685
Year: 2016-2020
Season: first season
RMSE = 1.12
R^2 = 0.44
N_kg_ha
n = 326
p < 0.0001
≤ 27
> 27
hybrid
n = 243
p < 0.0001
no
yes
season_prec
n = 202
p < 0.0001
≤ 637.8
> 637.8
season_prec
n = 136
p = 9e-04
≤ 788.08
> 788.08
n = 66
n = 117
n = 19
n = 41
n = 83
2.04 Mg ha^{-1}
1.18 Mg ha^{-1}
1.81 Mg ha^{-1}
2.69 Mg ha^{-1}
3.7 Mg ha^{-1}
Yield (Mg ha^{-1})

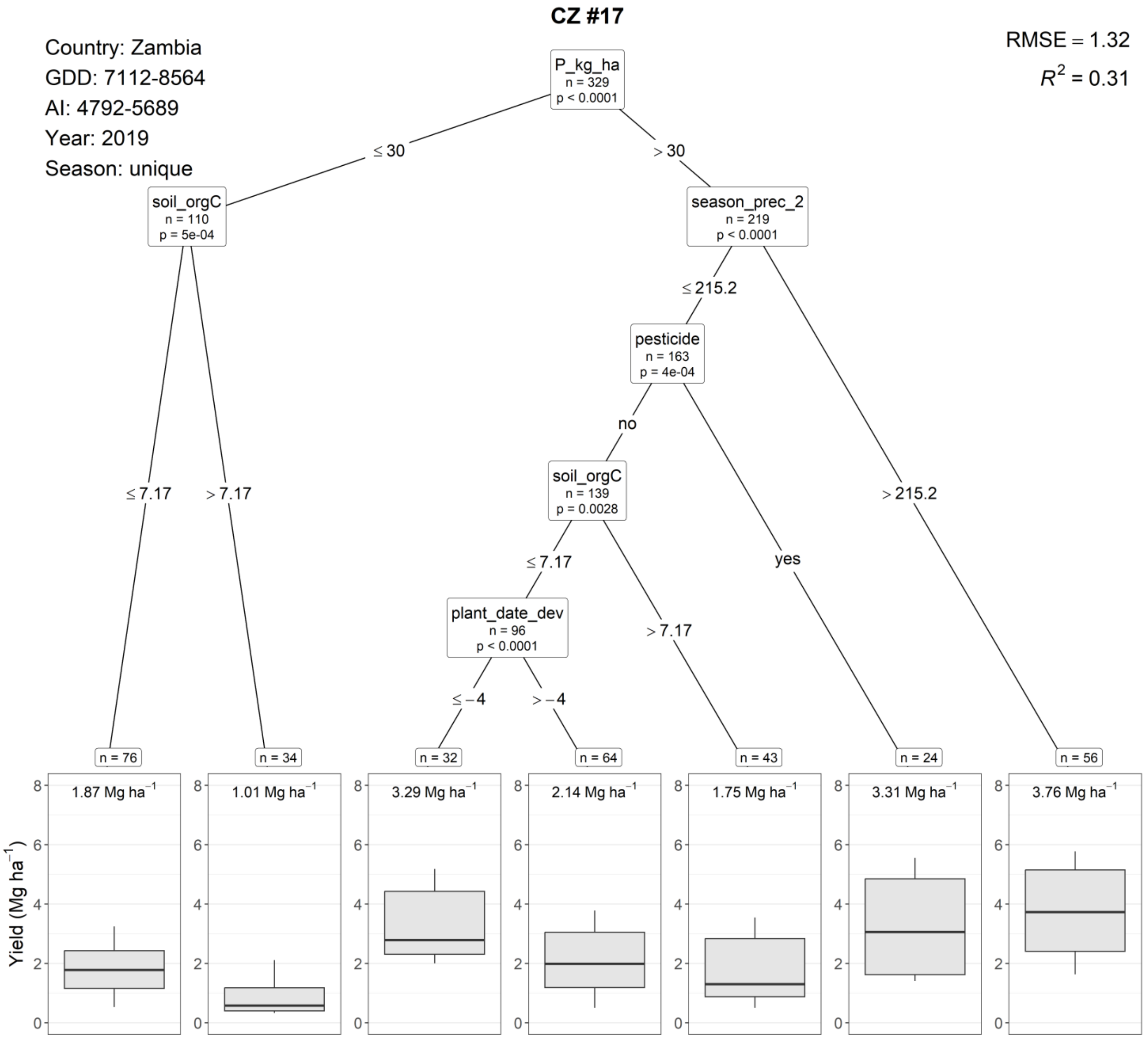
CZ #17
Country: Zambia
GDD: 7112-8564
AI: 4792-5689
Year: 2019
Season: unique
RMSE = 1.32
R^2 = 0.31
P_kg_ha
n = 329
p < 0.0001
≤ 30
> 30
soil_orgC
n = 110
p = 5e-04
season_prec_2
n = 219
p < 0.0001
≤ 215.2
> 215.2
pesticide
n = 163
p = 4e-04
no
yes
soil_orgC
n = 139
p = 0.0028
≤ 7.17
> 7.17
≤ 7.17
> 7.17
plant_date_dev
n = 96
p < 0.0001
≤ −4
> −4
n = 76
n = 34
n = 32
n = 64
n = 43
n = 24
n = 56
1.87 Mg ha^{-1}
1.01 Mg ha^{-1}
3.29 Mg ha^{-1}
2.14 Mg ha^{-1}
1.75 Mg ha^{-1}
3.31 Mg ha^{-1}
3.76 Mg ha^{-1}
Yield (Mg ha^{-1})
0
2
4
6
8

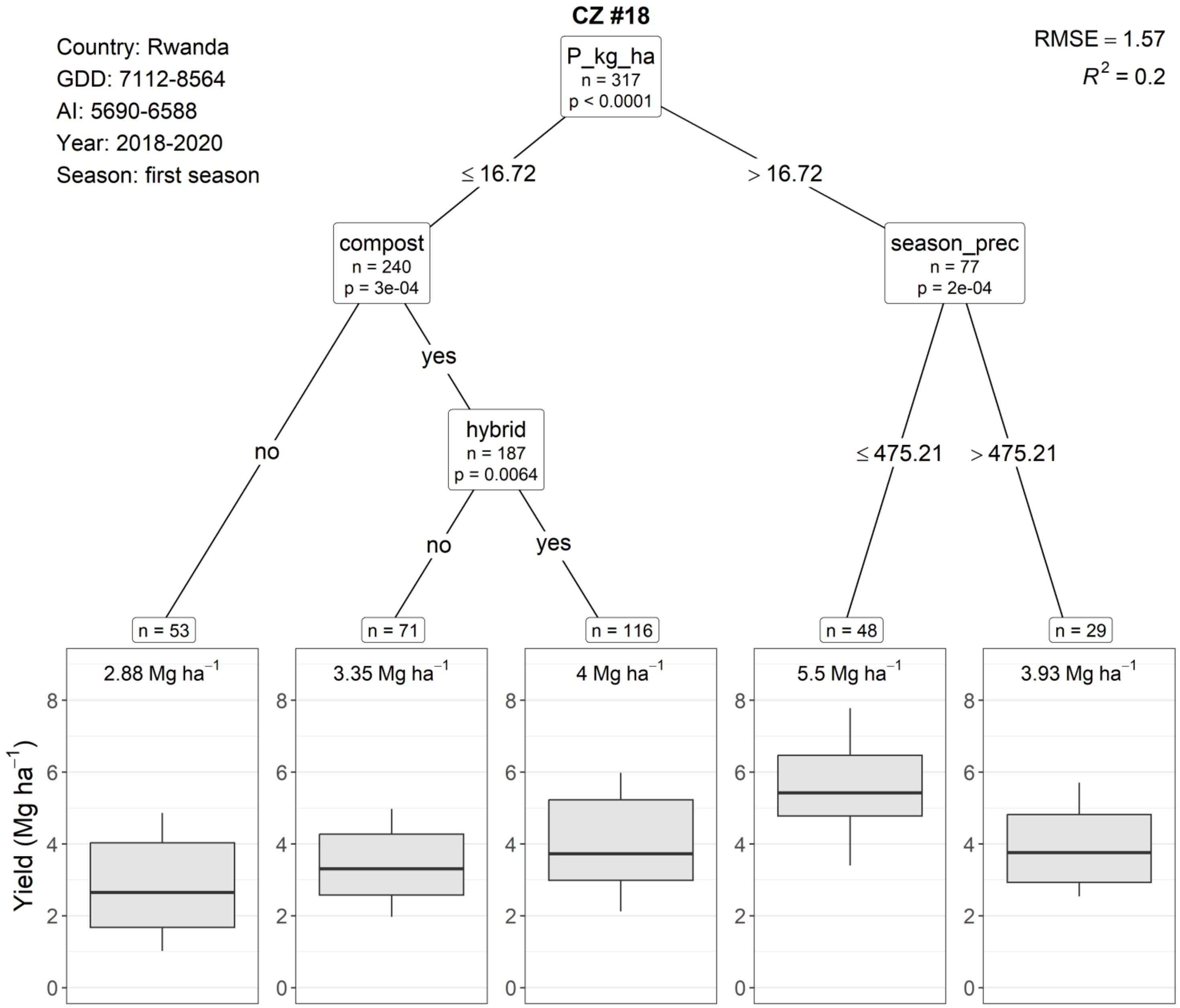
CZ #18
Country: Rwanda
GDD: 7112-8564
AI: 5690-6588
Year: 2018-2020
Season: first season
RMSE = 1.57
R^2 = 0.2
P_kg_ha
n = 317
p < 0.0001
≤ 16.72
> 16.72
compost
n = 240
p = 3e-04
season_prec
n = 77
p = 2e-04
no
yes
hybrid
n = 187
p = 0.0064
no
yes
≤ 475.21
> 475.21
n = 53
n = 71
n = 116
n = 48
n = 29
2.88 Mg ha^{-1}
3.35 Mg ha^{-1}
4 Mg ha^{-1}
5.5 Mg ha^{-1}
3.93 Mg ha^{-1}
Yield (Mg ha^{-1})
0
2
4
6
8

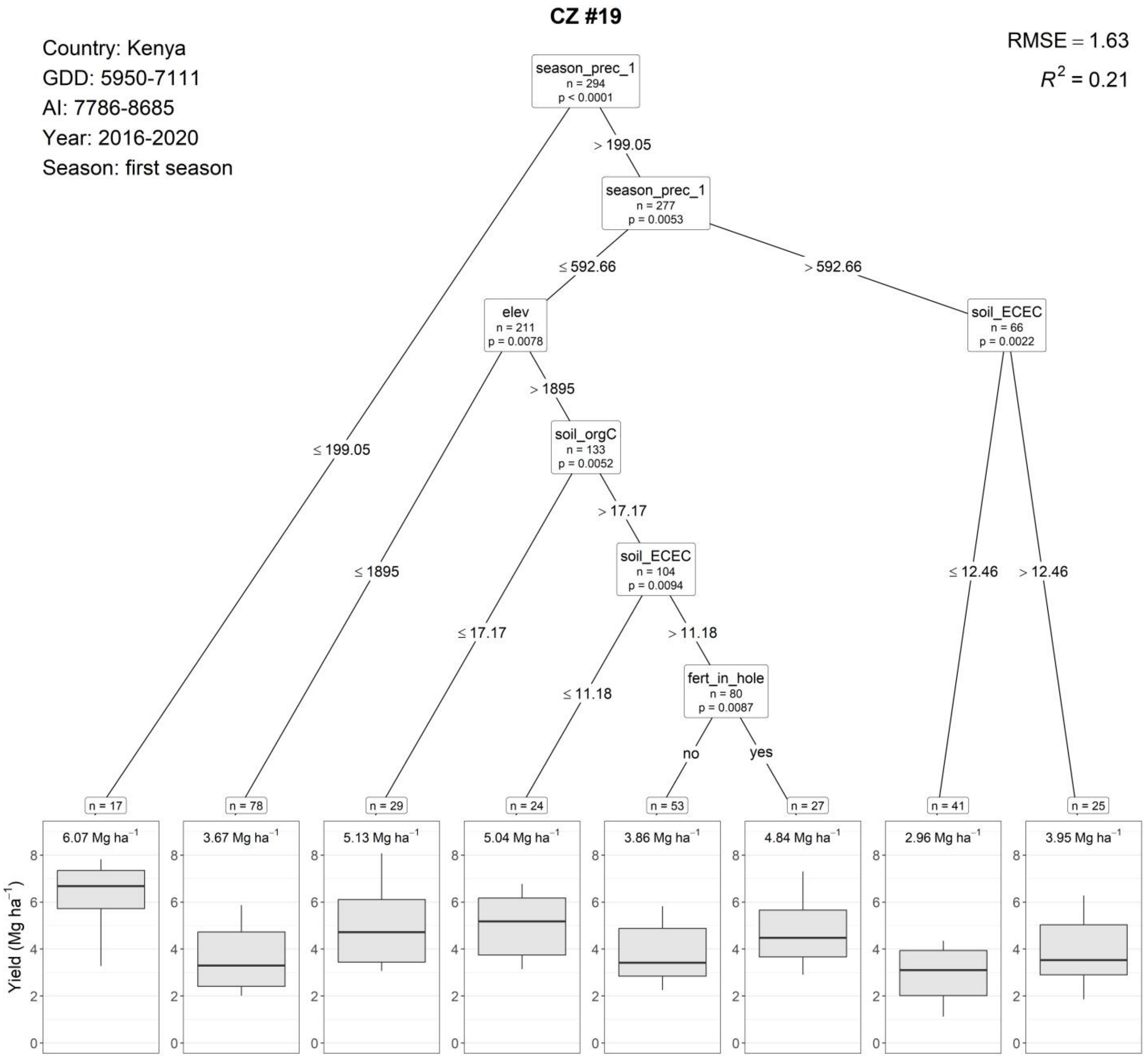

CZ #19
Country: Kenya
GDD: 5950-7111
AI: 7786-8685
Year: 2016-2020
Season: first season
RMSE = 1.63
R^2 = 0.21
season_prec_1
n = 294
p < 0.0001
> 199.05
≤ 199.05
season_prec_1
n = 277
p = 0.0053
≤ 592.66
> 592.66
elev
n = 211
p = 0.0078
> 1895
≤ 1895
soil_orgC
n = 133
p = 0.0052
> 17.17
≤ 17.17
soil_ECEC
n = 104
p = 0.0094
≤ 11.18
> 11.18
fert_in_hole
n = 80
p = 0.0087
no
yes
soil_ECEC
n = 66
p = 0.0022
≤ 12.46
> 12.46
n = 17
n = 78
n = 29
n = 24
n = 53
n = 27
n = 41
n = 25
6.07 Mg ha^{-1}
3.67 Mg ha^{-1}
5.13 Mg ha^{-1}
5.04 Mg ha^{-1}
3.86 Mg ha^{-1}
4.84 Mg ha^{-1}
2.96 Mg ha^{-1}
3.95 Mg ha^{-1}
Yield (Mg ha^{-1})

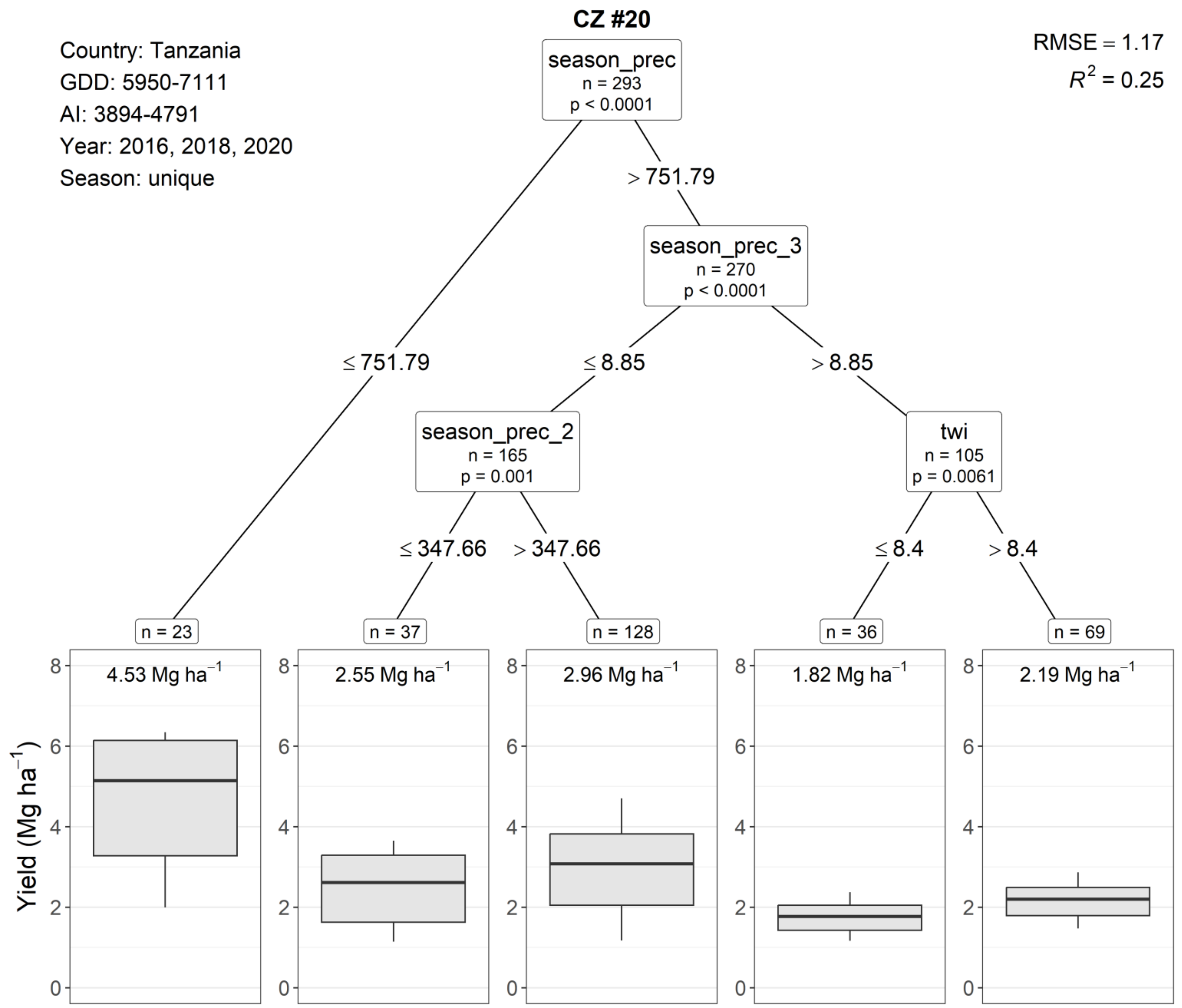
CZ #20
Country: Tanzania
GDD: 5950-7111
AI: 3894-4791
Year: 2016, 2018, 2020
Season: unique
RMSE = 1.17
R^2 = 0.25
season_prec
n = 293
p < 0.0001
≤ 751.79
> 751.79
season_prec_3
n = 270
p < 0.0001
≤ 8.85
> 8.85
season_prec_2
n = 165
p = 0.001
twi
n = 105
p = 0.0061
≤ 347.66
> 347.66
≤ 8.4
> 8.4
n = 23
n = 37
n = 128
n = 36
n = 69
4.53 Mg ha^{-1}
2.55 Mg ha^{-1}
2.96 Mg ha^{-1}
1.82 Mg ha^{-1}
2.19 Mg ha^{-1}
Yield (Mg ha^{-1})

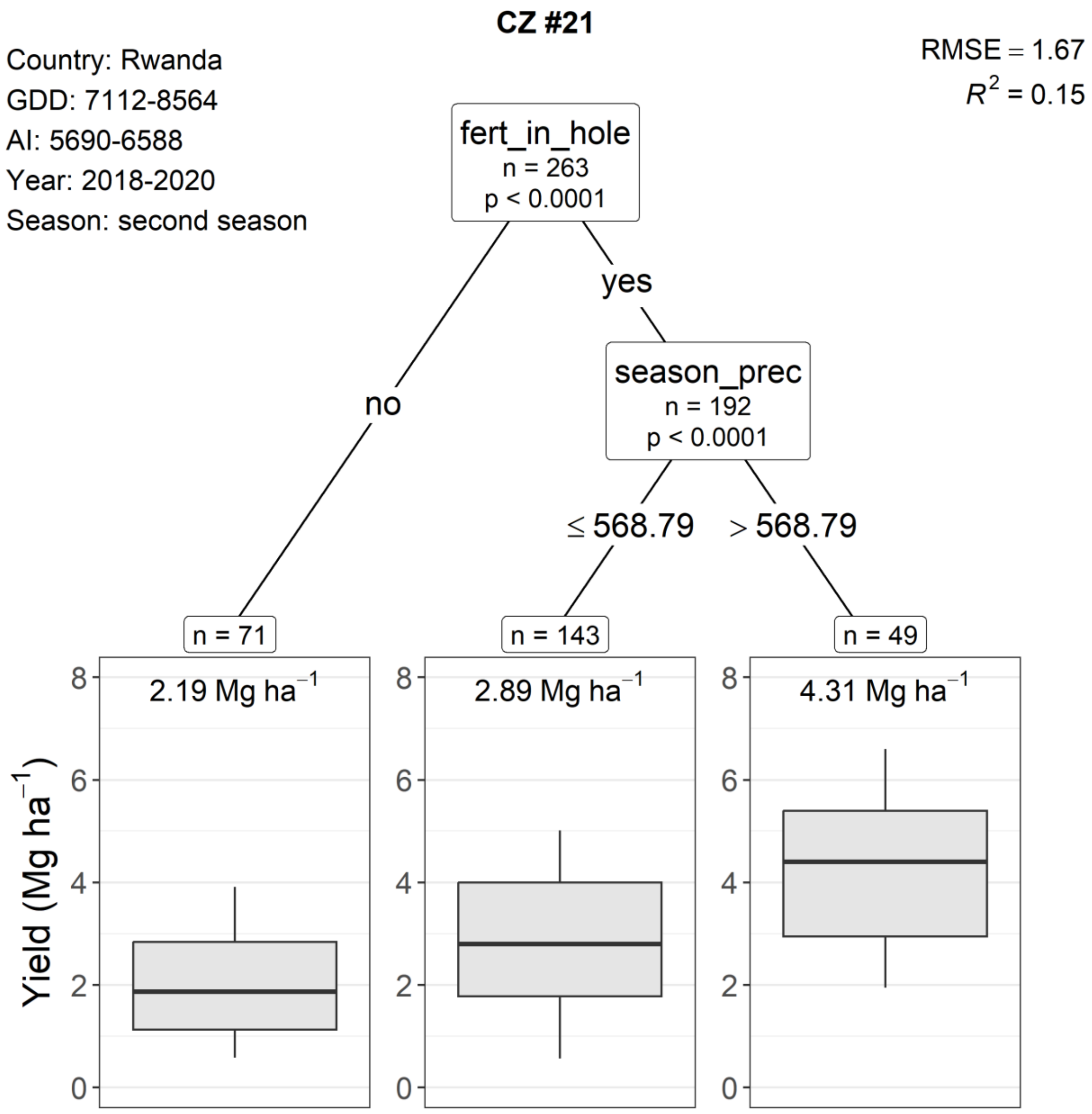
CZ #21
Country: Rwanda
GDD: 7112-8564
AI: 5690-6588
Year: 2018-2020
Season: second season
RMSE = 1.67
R^2 = 0.15
fert_in_hole
n = 263
p < 0.0001
no
yes
season_prec
n = 192
p < 0.0001
≤ 568.79
> 568.79
n = 71
n = 143
n = 49
2.19 Mg ha^{-1}
2.89 Mg ha^{-1}
4.31 Mg ha^{-1}
Yield (Mg ha^{-1})
0
2
4
6
8

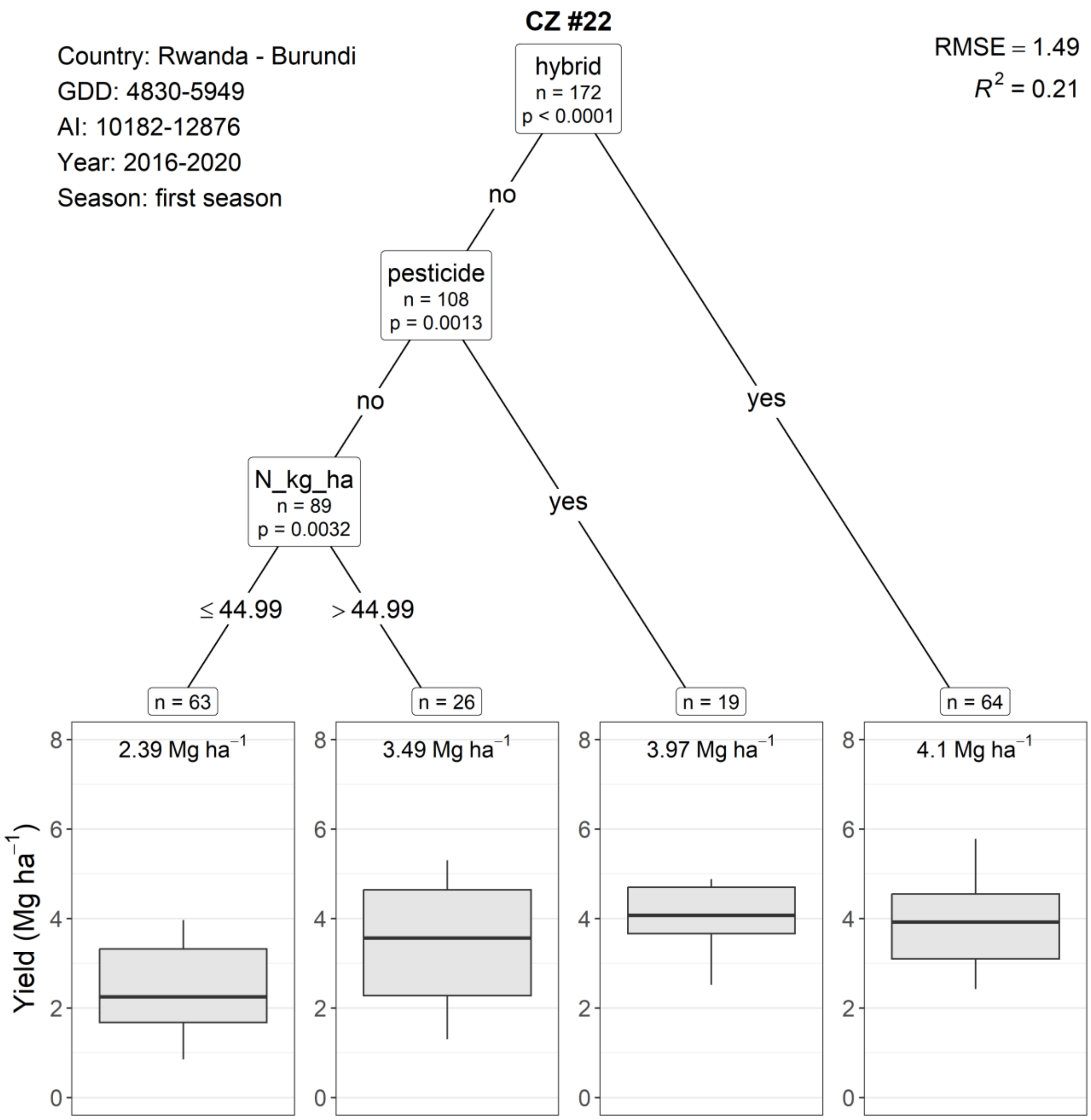
CZ #22
Country: Rwanda - Burundi
GDD: 4830-5949
AI: 10182-12876
Year: 2016-2020
Season: first season
RMSE = 1.49
R2 = 0.21
hybrid
n = 172
p < 0.0001
no
yes
pesticide
n = 108
p = 0.0013
no
yes
N_kg_ha
n = 89
p = 0.0032
≤ 44.99
> 44.99
n = 63
n = 26
n = 19
n = 64
2.39 Mg ha-1
3.49 Mg ha-1
3.97 Mg ha-1
4.1 Mg ha-1
Yield (Mg ha-1)
0
2
4
6
8

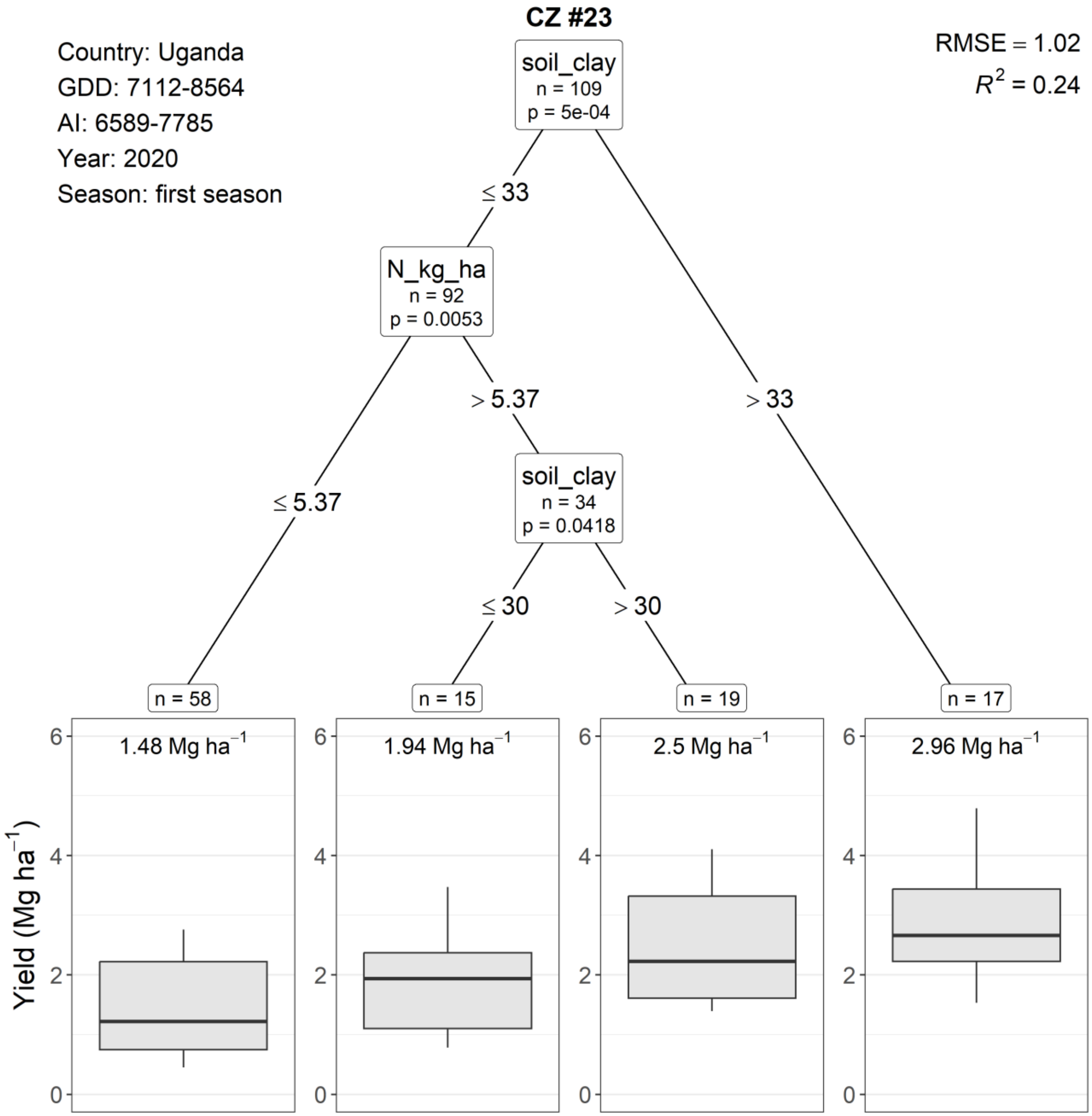
CZ #23
Country: Uganda
GDD: 7112-8564
AI: 6589-7785
Year: 2020
Season: first season
RMSE = 1.02
R2 = 0.24
soil_clay
n = 109
p = 5e-04
≤ 33
> 33
N_kg_ha
n = 92
p = 0.0053
≤ 5.37
> 5.37
soil_clay
n = 34
p = 0.0418
≤ 30
> 30
n = 58
n = 15
n = 19
n = 17
1.48 Mg ha−1
1.94 Mg ha−1
2.5 Mg ha−1
2.96 Mg ha−1
Yield (Mg ha−1)
0
2
4
6

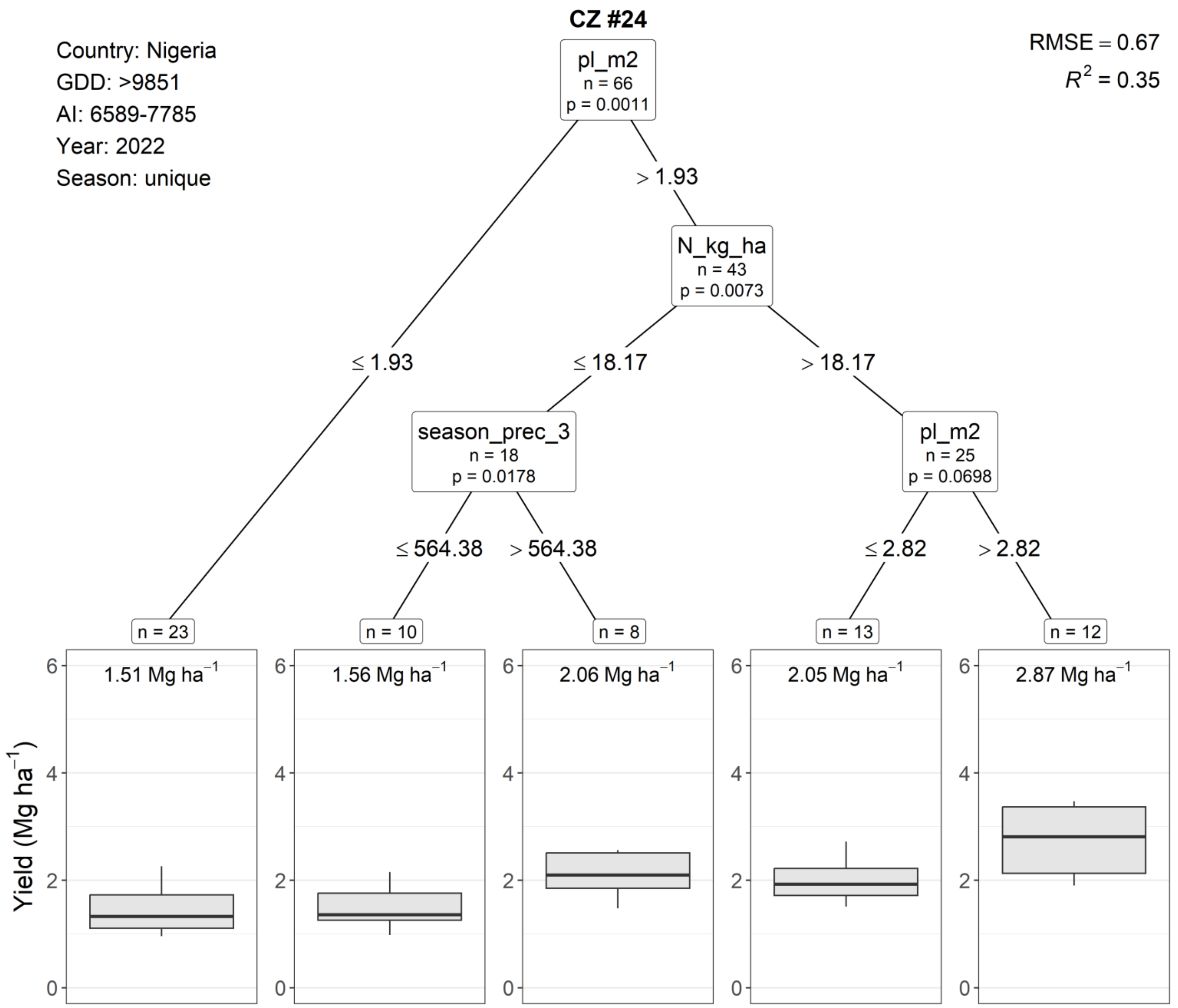
CZ #24
Country: Nigeria
GDD: >9851
AI: 6589-7785
Year: 2022
Season: unique
RMSE = 0.67
R^2 = 0.35
pl_m2
n = 66
p = 0.0011
> 1.93
≤ 1.93
N_kg_ha
n = 43
p = 0.0073
≤ 18.17
> 18.17
season_prec_3
n = 18
p = 0.0178
pl_m2
n = 25
p = 0.0698
≤ 564.38
> 564.38
≤ 2.82
> 2.82
n = 23
n = 10
n = 8
n = 13
n = 12
1.51 Mg ha^{-1}
1.56 Mg ha^{-1}
2.06 Mg ha^{-1}
2.05 Mg ha^{-1}
2.87 Mg ha^{-1}
Yield (Mg ha^{-1})
0
2
4
6

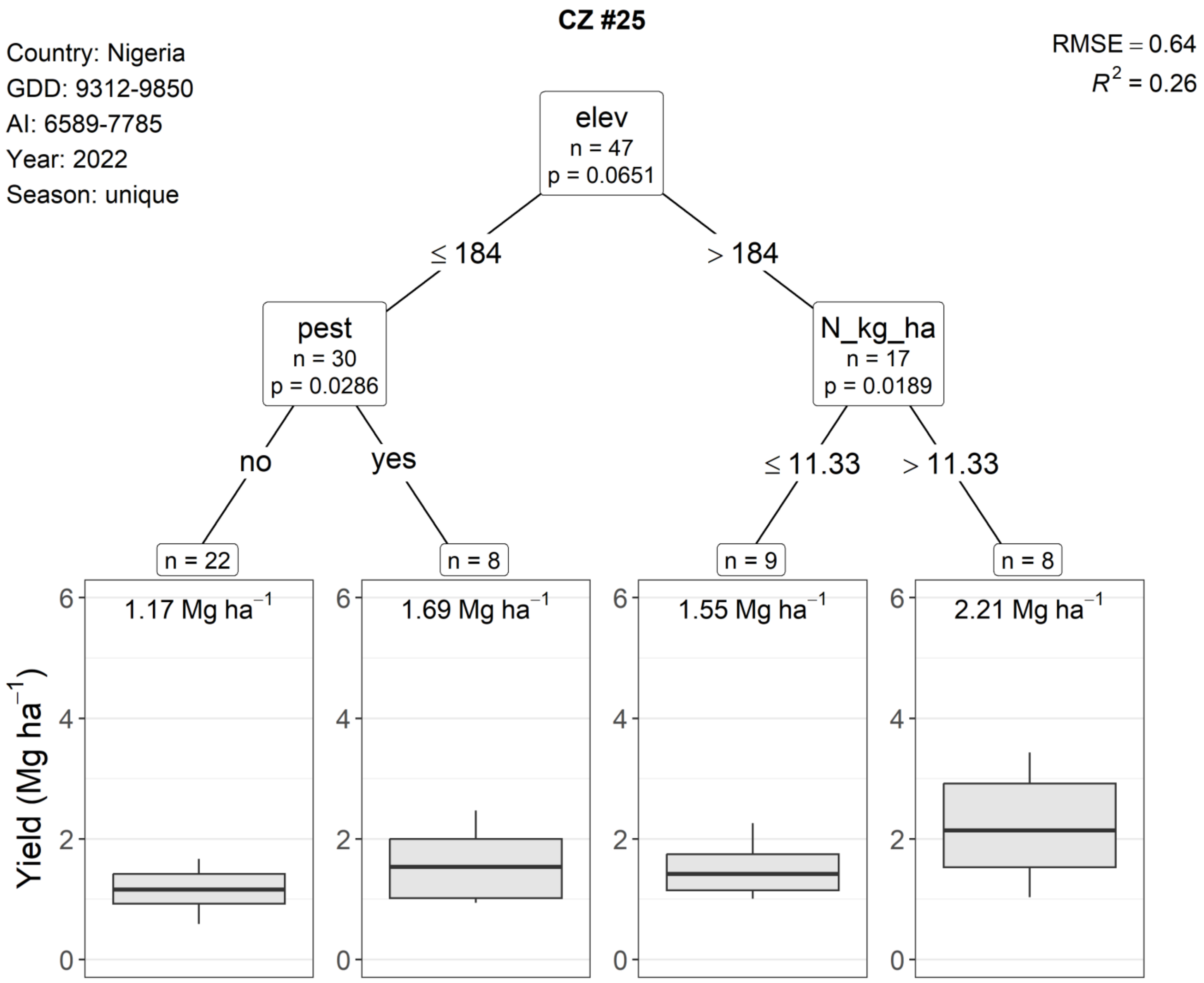
CZ #25
Country: Nigeria
GDD: 9312-9850
AI: 6589-7785
Year: 2022
Season: unique
RMSE = 0.64
R^2 = 0.26
elev
n = 47
p = 0.0651
≤ 184
> 184
pest
n = 30
p = 0.0286
N_kg_ha
n = 17
p = 0.0189
no
yes
≤ 11.33
> 11.33
n = 22
n = 8
n = 9
n = 8
1.17 Mg ha^{-1}
1.69 Mg ha^{-1}
1.55 Mg ha^{-1}
2.21 Mg ha^{-1}
Yield (Mg ha^{-1})
0
2
4
6

Supplementary Code

Code used for the conditional inference tree analysis shown in Supplementary Figure 12. The code is also available on GitHub.

```
library(data.table) # version 1.14.8
library(partykit) # version 1.2.20

# prepare data -------------
# database available at https://zenodo.org/records/11122388
d <- fread("One_Acre_Fund_MEL_maize_survey_data_2016-2022.csv", na.strings = "")
d <- d[!is.na(strat), ]

strats <- d[, unique(strat)]

## prediction selection ---------
preds <- c(
  # seasonal weather
  "season_prec", paste0("season_prec_", 1:3),
  # elevation and topography
  "elev", "twi",
  # soil properties
  "soil_rzpawhc", "soil_clay", "soil_pH","soil_orgC", "soil_ECEC",
  # planting management and cultivar
  "plant_date_dev", "pl_m2", "row_spacing",
  # cultivar
  "hybrid", "hyb_mat", "hyb_type", "hyb_yor", "hyb_tol_mln", "hyb_tol_msv",
  "hyb_tol_gls", "hyb_tol_nclb", "hyb_tol_rust", "hyb_tol_ear_rot",
  # nutrient management
  "N_kg_ha", "P_kg_ha", "K_kg_ha",
  "compost", "comp_kg_ha", "manure",
  "fert_in_hole",
  # liming
  "lime_kg_ha",
  # crop management
  "weeding", "pesticide",
  # adversities
  "disease", "pest", "striga", "water_excess"
)

ypreds <- c("yield_kg_ha", preds)
```

```
## logical to yes/no -----------------------
for(j in preds[sapply(d[,..preds], is.logical)]) {
  set(d, i = NULL, j, as.character(d[[j]]))
  d[d[[j]] == "TRUE", (j):= "yes"]
  d[d[[j]] == "FALSE", (j):= "no"]
  set(d, i = NULL, j, as.factor(d[[j]]))
}

## change character to factor ----------
for(j in preds[sapply(d[,..preds], is.character)]) {
  set(d, i = NULL, j, as.factor(d[[j]]))
}

# Run trees -----------------------------------

# loop over CZ by country and season.
lt <- vector("list", length(strats))
names(lt) <- strats

for(i in seq_along(lt)) {

  ii <- d[, .I[strat == strats[i]]]
  di <- d[ii, ..ypreds]

  ## control for conditional inference trees
  ctrl <- ctree_control(
    teststat = "quadratic",
    testtype = "MonteCarlo",
    nresample = 1e4,
    mincriterion = .99,
    minsplit = min(round(di[,.N] * .20), 200),
    minbucket = max(min(round(di[,.N] * .05), 50), 16),
    maxdepth = 10
  )
  if(strats[i] %like% "UGA|NGA") {
    ctrl <- ctree_control(
      teststat = "quadratic",
      testtype = "MonteCarlo",
      nresample = 1e4,
      mincriterion = .90,
      minsplit = 10,
      minbucket = 8,
```

```
      maxdepth = 10
    )
  }

  # remove columns with not enough data
  # 25 % is needed for a split.
  for(j in preds) {
    j_NA_p <- sum(is.na(di[[j]]))
    if(j_NA_p > ctrl$minsplit) di[, (j):= NULL]
  }

  # 8% is needed in a terminal node.
  # So at least there should be 8% of incidence of an adversity to be considered.
  for(j in c("disease", "pest", "water_excess")) {
    j_T_p <- sum(di[[j]] == "yes") # note that logic vars were transformed to factor
    if(j_T_p < ctrl$minbucket) di[, (j):= NULL]
  }

  # fit model
  set.seed(0)
  lt[[i]] <- ctree(
    yield_kg_ha ~ .,
    data = di,
    control = ctrl
  )
}
```
