# Supplementary material for: Adopting yield-improving practices to meet maize demand in Sub-Saharan Africa without cropland expansion
Source: Nat Commun. 2024 May 27;15:4492. doi: 10.1038/s41467-024-48859-0 (PMC11130130; doi:10.1038/s41467-024-48859-0)
Supplement: Supplementary file 2 — Reporting Summary [file 41467_2024_48859_MOESM2_ESM.pdf]

nature portfolio

Corresponding author(s): Patricio Grassini

Last updated by author(s): May 7, 2024

Reporting Summary

Nature Portfolio wishes to improve the reproducibility of the work that we publish. This form provides structure for consistency and transparency in reporting. For further information on Nature Portfolio policies, see our Editorial Policies and the Editorial Policy Checklist.

Statistics

For all statistical analyses, confirm that the following items are present in the figure legend, table legend, main text, or Methods section.

n/a	Confirmed	
☐	☒	The exact sample size (*n*) for each experimental group/condition, given as a discrete number and unit of measurement
☐	☒	A statement on whether measurements were taken from distinct samples or whether the same sample was measured repeatedly
☐	☒	The statistical test(s) used AND whether they are one- or two-sided<br>*Only common tests should be described solely by name; describe more complex techniques in the Methods section.*
☐	☒	A description of all covariates tested
☐	☒	A description of any assumptions or corrections, such as tests of normality and adjustment for multiple comparisons
☐	☒	A full description of the statistical parameters including central tendency (e.g. means) or other basic estimates (e.g. regression coefficient) AND variation (e.g. standard deviation) or associated estimates of uncertainty (e.g. confidence intervals)
☐	☒	For null hypothesis testing, the test statistic (e.g. *F*, *t*, *r*) with confidence intervals, effect sizes, degrees of freedom and *P* value noted<br>*Give P values as exact values whenever suitable.*
☒	☐	For Bayesian analysis, information on the choice of priors and Markov chain Monte Carlo settings
☒	☐	For hierarchical and complex designs, identification of the appropriate level for tests and full reporting of outcomes
☒	☐	Estimates of effect sizes (e.g. Cohen's *d*, Pearson's *r*), indicating how they were calculated

Our web collection on statistics for biologists contains articles on many of the points above.

Software and code

Policy information about availability of computer code

Data collection	The field-level yield, management, and biophysical data generated in this study have been deposited in Zenodo (https://zenodo.org/doi/10.5281/zenodo.11115815).
Data analysis	All data analysis was conducted in R (version 4.2.1) with the following packages and versions: terra (1.6.17), data.table (1.14.2), stringr (1.4.0), car (3.1.0), partykit (1.2.16), ltm (1.2.0), emmeans (1.8.5), lme4 (1.1.30), xgboost (1.7.5.1), treeshap (0.3.0), and CAST (0.8.1).). The R code for the current study is publicly on GitHub: https://github.com/AramburuMerlos/SSA_maize_management

For manuscripts utilizing custom algorithms or software that are central to the research but not yet described in published literature, software must be made available to editors and reviewers. We strongly encourage code deposition in a community repository (e.g. GitHub). See the Nature Portfolio guidelines for submitting code & software for further information.

Data

Policy information about availability of data

All manuscripts must include a data availability statement. This statement should provide the following information, where applicable:

- Accession codes, unique identifiers, or web links for publicly available datasets
- A description of any restrictions on data availability
- For clinical datasets or third party data, please ensure that the statement adheres to our policy

The field-level yield, management, and biophysical data generated in this study have been deposited in Zenodo (https://zenodo.org/doi/10.5281/zenodo.11115815) 75. Data on yield potential from Global Yield Gap Atlas are available at www.yieldgap.org. Data on national average maize yield, harvested area, production, export,

and import, and demand from FAOSTAT are available at www.fao.org/faostat. Data on maize distribution from SPAM map are available in www.mapspam.info. Data on population size from UN are available at https://population.un.org/wpp/. Data on per-capita future maize demand is available at https://www.ifpri.org/project/ifpri-impact-model. Spatial vector data with administrative borders is available at https://gadm.org/. Source data are provided with this paper.

Research involving human participants, their data, or biological material

Policy information about studies with human participants or human data. See also policy information about sex, gender (identity/presentation), and sexual orientation and race, ethnicity and racism.

Reporting on sex and gender	No sex or gender data was recorded.
Reporting on race, ethnicity, or other socially relevant groupings	No socially constructed or socially relevant categorization variable was used in our manuscript.
Population characteristics	No population characteristics of the human research participants were collected or used.
Recruitment	Maize fields were selected based on farmers affiliation to the One Acre Fund program or for being neighbors of a field cropped under such program. Maize fields under the One Acre Fund program are expected to have greater technology adoption. We see inclusion of fields with varying level of technology adoption in the database as an advantage as it allows to increase the variation in management practices across farmer fields.
Ethics oversight	The study protocol was approved by the University of Nebraska-Lincoln and One Acre Fund.

Note that full information on the approval of the study protocol must also be provided in the manuscript.

Field-specific reporting

Please select the one below that is the best fit for your research. If you are not sure, read the appropriate sections before making your selection.

☐ Life sciences ☐ Behavioural & social sciences ☒ Ecological, evolutionary & environmental sciences

For a reference copy of the document with all sections, see nature.com/documents/nr-reporting-summary-flat.pdf

Ecological, evolutionary & environmental sciences study design

All studies must disclose on these points even when the disclosure is negative.

Study description	We measured maize yields and final plant stands and collected data on agronomic practices via surveys to smallholder farmers to identify agronomic practices that impact maize productivity most. Observations were stratified through climate zones. We included 26 management practice variables and 14 environmental covariables in the analysis. The effect of these variables on maize yields was analyzed with conditional inference trees, linear mixed-effects models, and machine learning (Gradient Boosting Machines and SHAP).
Research sample	The research sample includes 14,773 maize fields (Zea mays) from smallholder farmers in Sub-Saharan Africa. The sample was chosen to include fields with varying levels of technology adoption. The sample represents the various technologies smallholders used in maize fields in Sub-Saharan Africa.
Sampling strategy	Data was collected from maize fields following the One Acre Fund program (whose farmers were willing to complete the survey) and from neighboring fields that did not follow the program. The sample size from neighboring fields was chosen to match the fields following the program. Data was stratified via climate zones. Only data from climate zones with more than 200 observations were used to ensure a sufficient sample size.
Data collection	In each field, One Acre Fund Monitoring and Evaluation (OAF MEL) team members measured maize grain yield, plant density, and row spacing in two randomly placed boxes of 36 m2 at harvest, avoiding field edges. Data on agronomic management practices were collected through farmers surveys. The questions were provided to OAF MEL team members on a digital device. OAF MEL team asked them orally and recorded the answers from farmers in this device.
Timing and spatial scale	Data was collected every year between 2016 and 2022 from seven countries in Sub-Saharan Africa: Burundi, Kenya, Nigeria, Rwanda, Tanzania, Uganda, and Zambia.
Data exclusions	We excluded observations identified as yield outliers, as detected with a Bonferroni Outlier Test. Observations with plant densities or fertilizer rates higher than four standard deviations from the mean and those from atypical sowing dates were also excluded. The final dataset only included observations with at least geolocation, yield, and fertilizer data and for those climate zones with more than 200 observations. All exclusion criteria were established before performing any statistical analysis on the effect of agronomic practices on maize yields.
Reproducibility	The field-level yield, management, and biophysical data generated in this study have been deposited in Zenodo (https://zenodo.org/doi/10.5281/zenodo.11115815).
Randomization	Not applicable as we did not conduct field experiments.

Blinding	Not applicable.

Did the study involve field work? [x] Yes [ ] No

Field work, collection and transport

Field conditions	Measurements were conducted at harvest on farmers fields, at the end of the rainy season. All maize fields were rainfed (not irrigated).
Location	Samples were collected from 14,773 maize fields in Sub-Saharan Afruca in seven countries Burundi, Kenya, Nigeria, Rwanda, Tanzania, Uganda, and Zambia.
Access & import/export	Not applicable
Disturbance	Not applicable

Reporting for specific materials, systems and methods

We require information from authors about some types of materials, experimental systems and methods used in many studies. Here, indicate whether each material, system or method listed is relevant to your study. If you are not sure if a list item applies to your research, read the appropriate section before selecting a response.

Materials & experimental systems

n/a	Involved in the study	
[x]	[ ]	Antibodies
[x]	[ ]	Eukaryotic cell lines
[x]	[ ]	Palaeontology and archaeology
[x]	[ ]	Animals and other organisms
[x]	[ ]	Clinical data
[x]	[ ]	Dual use research of concern
[ ]	[x]	Plants

Methods

n/a	Involved in the study	
[x]	[ ]	ChIP-seq
[x]	[ ]	Flow cytometry
[x]	[ ]	MRI-based neuroimaging
